# Partitioning Phenotypic Variance Due to Parent-of-Origin Effects Using Genomic Relatedness Matrices

**DOI:** 10.1007/s10519-017-9880-0

**Published:** 2017-11-02

**Authors:** Charles Laurin, Gabriel Cuellar-Partida, Gibran Hemani, George Davey Smith, Jian Yang, David M. Evans

**Affiliations:** 1 MRC Integrative Epidemiology Unit, University of Bristol, Bristol, UK; 2 Faculty of Medicine, Translational Research Institute, The University of Queensland Diamantina Institute, Brisbane, QLD Australia; 3 Institute for Molecular Bioscience and Queensland Brain Institute, The University of Queensland, Brisbane, QLD Australia

**Keywords:** Imprinting, Parent-of-origin effects, ALSPAC, G-REML, GCTA

## Abstract

We propose a new method, *G-REMLadp*, to estimate the phenotypic variance explained by parent-of-origin effects (POEs) across the genome. Our method uses restricted maximum likelihood analysis of genome-wide genetic relatedness matrices based on individuals’ phased genotypes. Genome-wide SNP data from parent child duos or trios is required to obtain relatedness matrices indexing the parental origin of offspring alleles, as well as offspring phenotype data to partition the trait variation into variance components. To calibrate the power of *G-REMLadp* to detect non-null POEs when they are present, we provide an analytic approximation derived from Haseman-Elston regression. We also used simulated data to quantify the power and Type I Error rates of *G-REMLadp*, as well as the sensitivity of its variance component estimates to violations of underlying assumptions. We subsequently applied *G-REMLadp* to 36 phenotypes in a sample of individuals from the Avon Longitudinal Study of Parents and Children (ALSPAC). We found that the method does not seem to be inherently biased in estimating variance due to POEs, and that substantial correlation between parental genotypes is necessary to generate biased estimates. Our empirical results, power calculations and simulations indicate that sample sizes over 10000 unrelated parent-offspring duos will be necessary to detect POEs explaining < 10% of the variance with moderate power. We conclude that POEs tagged by our genetic relationship matrices are unlikely to explain large proportions of the phenotypic variance (i.e. > 15%) for the 36 traits that we have examined.

**Electronic supplementary material:**

The online version of this article (doi:10.1007/s10519-017-9880-0) contains supplementary material, which is available to authorized users.

## Introduction

Parent-of-origin effects (POEs) describe the phenomenon in which the effects of alleles depend upon their parental origin. POEs imply that heterozygote individuals have phenotypes which are distributed differently depending upon which of their alleles were maternally and paternally transmitted (Guilmatre and Sharp 2012; Lawson et al. 2013). The extreme case of POEs is *polar overdominance*, where the two heterozygotes’ phenotypes differ in distribution but the two homozygotes share the same distribution (Hoggart et al. 2014). Imprinting, a phenomenon in which one parent’s allele is not expressed, is probably the most widely studied example of POE (Peters 2014).

POEs have traditionally been examined in the context of development, where, in mouse models, they have been associated with body size and social behavior (Peters 2014). One evolutionary explanation of POEs concerns genomic conflict between maternal and paternal genes in offspring, with paternal genes encouraging growth and solicitation of maternal care, even at the expense of the mother’s health, while maternal alleles are orientated toward success of all offspring, which do not necessarily share paternity (Patten et al. 2014). Other evolutionary explanations for POEs include: different territorial patterns in males and females and coadaptation of maternal and offspring genomes to maximize the efficiency of nurturing behaviors like suckling and grooming (Peters 2014).

Whilst there is considerable support for the importance of POEs in animals (Neugebauer et al. 2010; Lawson et al. 2013), evidence for the existence of POEs in the etiology of complex human traits and diseases is mixed, in part due to the relative paucity of genomic data from families (Kong et al. 2009; Guilmatre and Sharp 2012). Before the genomics era, the children-of-twins design (Nance and Corey 1976), pedigree analyses (Hall 1990), and parent-offspring regressions (Clemons 2000) provided some limited evidence for the existence of POEs in human populations. These approaches were not often able to distinguish parental effects (indirect effects of the parental genotype on offspring phenotype) from POEs (interaction between the sex of the transmitting parent and the direct allelic effect in offspring) (Hager et al. 2008). More recently, genome-wide association studies incorporating parent-of-origin information have been used to identify POEs at individual loci for age at menarche (Perry et al. 2014), Type I diabetes (Wallace et al. 2010), Type II diabetes, basal cell carcinoma, and breast cancer [all identified in (Kong et al. 2009)].

We propose a method, *G-REMLadp*, to estimate the phenotypic variance due to POEs across the genome by applying restricted maximum likelihood (REML) to offspring genome-wide genetic relatedness matrices and phenotype data. Our method involves the construction of a genetic relationship matrix indexing the parental origin of offspring alleles using genome-wide SNP data from parent–child duos or trios. The proposed method is an early adaptation to human genetics of procedures developed for animal breeding (Schaeffer et al. 1989; Spencer 2002; Nishio and Satoh 2015). The genotypic coding for POEs, which we adopt here, is based on the model outlined by Spencer (2002, 2009) and implemented by Nishio and Satoh (2015) in the context of genomic prediction. The genotype coding we used for dominance effects is due to Zhu et al. (2015), who designed it to be orthogonal to the allele count at a locus; it is also orthogonal to the POE coding, which distinguishes our approach from that used by Spencer, Nishio and Satoh, and others. Using these codings, the phased genotype at a locus is coded using three orthogonal components: an additive-coded genotype, a dominance-coded genotype, and a POE-coded genotype.

To estimate the power of *G-REMLadp* to estimate non-null POEs, we provide an approximation using Haseman–Elston regression (Elston et al. 2000; Chen 2014). We also used simulated data to estimate the power and Type I Error rates of *G-REMLadp*, as well as the sensitivity of its variance component estimates to violations of assumptions. We then applied *G-REMLadp* to 36 phenotypes related to body size and obesity, metabolic traits, and IQ in a sample of up to 4753 individuals from a UK-based longitudinal study of childhood health and development.

## Methods

*G-REMLadp* uses REML to fit a linear mixed model incorporating random additive effects, dominance effects, and POEs to human genomic data, with the goal of partitioning phenotypic variance into components reflecting these sources of variation tagged by genome-wide SNP chips. In this model, at each locus, the phased genotype of each individual $\left( {X \in aa,\,{a_{mo}}{A_{fa}},\,{A_{mo}}{a_{fa}},\,AA} \right)$ is expressed as 3 orthogonal components, which are then standardized. The codings for each of the 3 components are listed in Table 1. The standardized additive-coded genotype is the standardized minor allele (A) count at the locus. The derivation of the standardized dominance-coded genotype is given in Zhu et al. (2015); and a flexible equivalent coding is given in Álvarez-Castro (2014). The (unstandardized) POE-coded genotype is -1 for the paternal-minor heterozygote (${a_{mo}}{A_{fa}}$), where ${a_{mo}}$ indicates that the major allele was inherited from the mother and ${A_{fa}}$ that the minor allele was inherited from the father, and 1 for the maternal-minor heterozygote (${A_{mo}}{a_{fa}}$), and 0 for both homozygotes.

**Table 1:** Recoding a phased genotype using three orthogonal terms

Phased genotype	Freq	Add Code	Std add	Dom code	Std dom	POE code	Std POE
${a_{mo}}{a_{fa}}$	${q^2}$	0	$- \sqrt {2p/q}$	0	$- p/q$	0	0
${a_{mo}}{A_{fa}}$	$pq$	1	$\left( {q - p} \right)/\sqrt {2pq}$	$2p$	1	− 1	$- \,1/\sqrt {2pq}$
${A_{mo}}{a_{fa}}$	$pq$	1	$\left( {q - p} \right)/\sqrt {2pq}$	$2p$	1	1	$1/\sqrt {2pq}$
${A_{mo}}{A_{fa}}$	${p^2}$	2	$\sqrt {2q/p}$	$4p - 2$	$- q/p$	0	0

The minor allele is $A$, with frequency $p$, major allele $a$ with frequency $q=1 - p$. ${A_{mo}}$: maternally inherited $A$ allele; ${A_{fa}}$: paternally inherited $A$ allele. *“Freq”* expected frequency of the genotype under Hardy–Weinberg equilibrium. *“Add Code”* additive coding of the phased genotype. *“Std Add”* standardized additive coding based on mean $2p$ and variance $2pq$. *“Dom Code”* dominance coding of the phased genotype. *“Std Dom”* standardized dominance coding based on mean $2{p^2}$ and variance $4{p^2}{q^2}$. *“POE Code”* parent-of-origin effect coding of the phased genotype. *“Std POE”* standardized parent-of-origin effect coding based on mean $0$ and variance $2pq$

**Fig. 1:**
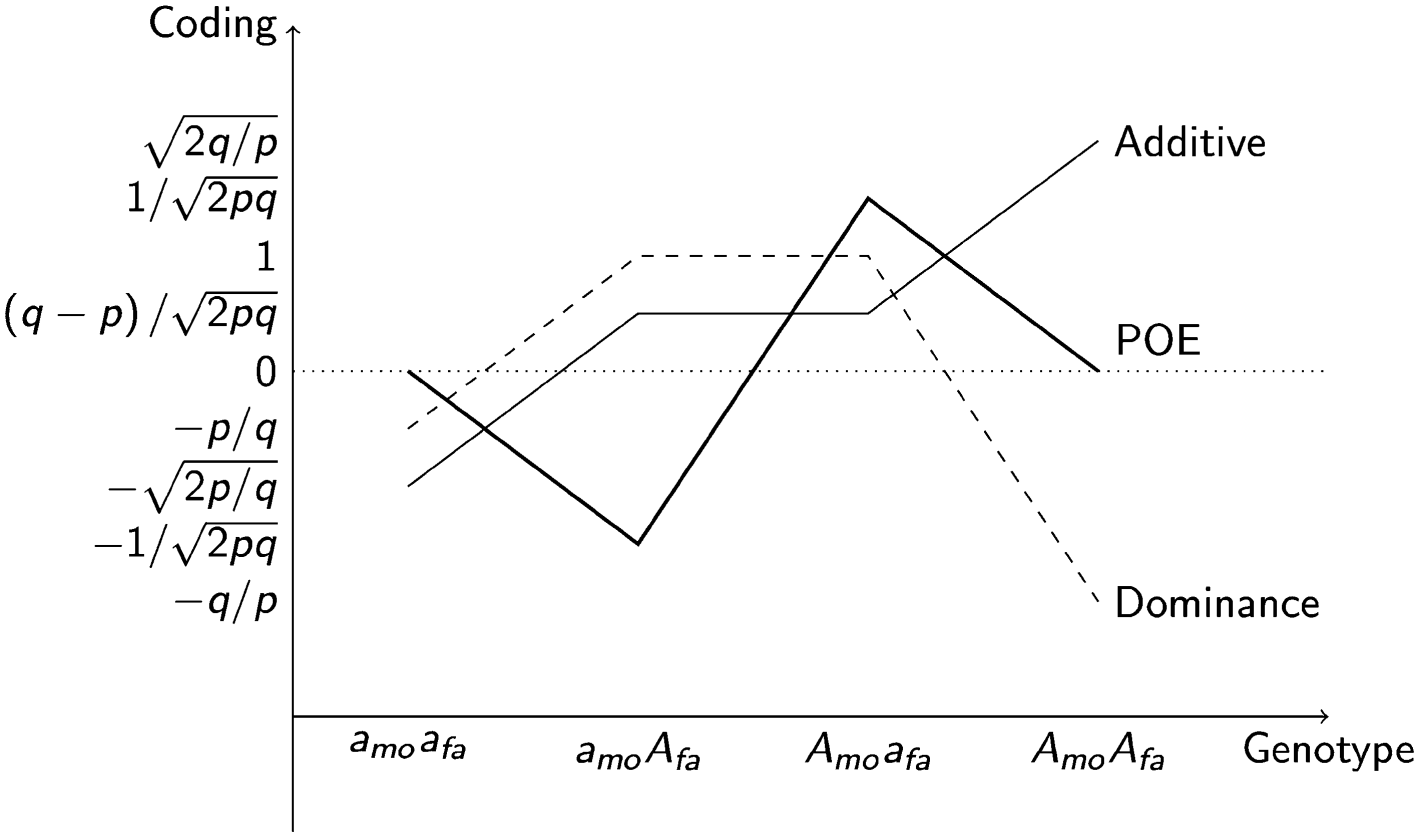
Phased genotype represented by 3 standardised codings: This figure shows the values of each of three effect-based codings for a given phased genotype, where *a* represents the major allele and *A* the minor (effect allele). The subscript *mo* indicates that the allele was transmitted maternally, while the subscript *fa* indicates paternal transmission. For a single locus with minor allele frequency $p$, the standardised additive coding (0, 1, 2 standardised to $- \sqrt {2p/q} ,\,\left( {q - p} \right)/\sqrt {2pq} ,\,\sqrt {2q/p}$) of the phased genotype is given by the medium-weight line. The standardised dominance coding is the dashed line ($0,2p,4p - 2$ standardised to$- p/q,\,1,\, - q/p$). The parent-of-origin effect coding is the thick line (0,− 1,1,0 standardised to $0,\, - 1/\sqrt {2pq} ,\,1/\sqrt {2pq} ,\,0$)

The mixed model utilizing these codings is given in Eq. 1, where ${\mathbf{y}}$ is a vector of phenotypes; ${{\mathbf{\mu }}_y}$is a column vector of means; the vector ${\mathbf{Tb}}$ includes any fixed effects of covariates ${\mathbf{T}}$; the ${\mathbf{Z}}$s are $n \times m$ matrices containing the standardised coded genotypes (indexing additive, dominance and POEs), represented as one individual per row (sample size $n$) and one SNP per column ($m$ markers affect the phenotype; in empirical data, this will be replaced by ${m_o}$, the number of observed markers, while in power analyses, this will be replaced by ${m_e}$, the number of effective markers); the ${\mathbf{\beta }}$ are $m$ × 1 column vectors of additive effects (*β*_*α*_), dominance effects (*β*_*δ*_) and POEs (*β*_*γ*_), which are assumed to be independently normally distributed with mean 0 and variances that are inversely proportional to the number of markers affecting the phenotype: ${\text{Var}}\left( {{\beta _\alpha }} \right)={m^{ - 1}}\sigma _{\alpha }^{2}{{\mathbf{I}}_n}$ (${\mathbf{I}}$ is the $n \times n$ identity matrix), ${\text{Var}}\left( {{\beta _\delta }} \right)={m^{ - 1}}\sigma _{\delta }^{2}{{\mathbf{I}}_n}$, ${\text{Var}}\left( {{\beta _\gamma }} \right)={m^{ - 1}}\sigma _{\gamma }^{2}{{\mathbf{I}}_n}$, respectively, and ${\mathbf{\varepsilon }}$ is a normal error variable with mean 0 and variance $\sigma _{\varepsilon }^{2}{{\mathbf{I}}_n}$ that is independent of the other variables on the right hand side of the equation. Figure 1 illustrates how the phased genotype is represented as the three orthogonal standardized coded genotypes, while Eq. 1 gives the phenotypic means at each phased genotype.

1 $${\mathbf{y}}={{\mathbf{\mu }}_{\mathbf{y}}}+{\mathbf{Tb}}+{{\mathbf{Z}}_{\mathbf{\alpha }}}{{\mathbf{\beta }}_{\mathbf{\alpha }}}+{{\mathbf{Z}}_{\mathbf{\delta }}}{{\mathbf{\beta }}_{\mathbf{\Delta }}}+{{\mathbf{Z}}_{\mathbf{\gamma }}}{{\mathbf{\beta }}_{\mathbf{\gamma }}}+{\mathbf{\varepsilon }}$$

The primary concern of this paper is with the partitioning of phenotypic variance components according to Equations 2 and 3, which present the problem in vector and individual-based forms, respectively.

2 $$\begin{array}{*{20}{l}} {{\text{Var}}\left( {\mathbf{y}} \right)}&{=E\left( {\left( {{\mathbf{y}} - {{\mathbf{\mu }}_{\mathbf{y}}} - {\mathbf{Tb}}} \right)\left( {{\mathbf{y}} - {{\mathbf{\mu }}_{\mathbf{y}}} - {\mathbf{Tb}}} \right)'} \right)} \\ {}&{={m^{ - 1}}\sigma _{\alpha }^{2}{{\mathbf{Z}}_{\mathbf{\alpha }}}{{\mathbf{Z}}_{\mathbf{\alpha }}}'+{m^{ - 1}}\sigma _{\delta }^{2}{{\mathbf{Z}}_{\mathbf{\delta }}}{{\mathbf{Z}}_{\mathbf{\delta }}}'+{m^{ - 1}}\sigma _{\gamma }^{2}{{\mathbf{Z}}_{\mathbf{\gamma }}}{{\mathbf{Z}}_{\mathbf{\gamma }}}'+\sigma _{\varepsilon }^{2}{{\mathbf{I}}_n}} \end{array}$$

where the matrices ${\mathbf{ZZ}}'$ are $n \times n$ matrices giving the cross-products, across all loci, of individuals’ coded genotypes. The elements of ${\text{Var}}\left( {\mathbf{y}} \right)$ are given by

3 $$\begin{array}{*{20}{l}} {{{\left\{ {{\text{Var}}\left( {\mathbf{y}} \right)} \right\}}_{ii}}}&{={m^{ - 1}}\left( {\mathop \sum \limits_{{k=1}}^{m} \sigma _{\alpha }^{2}z_{{\alpha ,\,ik}}^{2}+\sigma _{\delta }^{2}z_{{\delta ,\,ik}}^{2}+\sigma _{\gamma }^{2}z_{{\gamma ,\,ik}}^{2}} \right)+\sigma _{\varepsilon }^{2}} \\ {}&{=\sigma _{\alpha }^{2}+\sigma _{\delta }^{2}+\sigma _{\gamma }^{2}+\sigma _{\varepsilon }^{2}} \\ {{{\left\{ {{\text{Var}}\left( {\mathbf{y}} \right)} \right\}}_{ij}}}&{={m^{ - 1}}\left( {\sigma _{\alpha }^{2}\mathop \sum \limits_{{k=1}}^{m} {z_{\alpha ,\,ik}}{z_{\alpha ,\,jk}}+\sigma _{\delta }^{2}\mathop \sum \limits_{{k=1}}^{m} {z_{\delta ,\,ik}}{z_{\delta ,\,jk}}+\sigma _{\gamma }^{2}\mathop \sum \limits_{{k=1}}^{m} {z_{\gamma ,\,ik}}{z_{\gamma ,\,jk}}} \right)} \\ {}&{=\sigma _{\alpha }^{2}{{\mathbf{A}}_{ij}}+\sigma _{\delta }^{2}{{\mathbf{\Delta }}_{ij}}+\sigma _{\gamma }^{2}{{\mathbf{\Gamma }}_{ij}}} \\ {{\text{Var}}\left( {\mathbf{y}} \right)}&{=\sigma _{\alpha }^{2}{\mathbf{A}}+\sigma _{\delta }^{2}{\mathbf{\Delta }}+\sigma _{\gamma }^{2}{\mathbf{\Gamma }}+\sigma _{\varepsilon }^{2}{{\mathbf{I}}_n}} \end{array}$$

The second equality in Eq. 3 is because the mean squares of each individual’s standardized genotype codings are expected to be 1 in the absence of inbreeding.

The bold-faced Greek characters on the final line of Eq. 3 are used to denote the genetic relationship matrices (GRMs) for the three coded genotypes; the additive GRM is ${\mathbf{A}}$, the dominance GRM ${\mathbf{\Delta }}$, and the POE-coded GRM is ${\mathbf{\Gamma }}$. These are averages over all effective markers of the sums of squares and cross-products of individuals’ standardized coded genotypes.

It is important to note that we have assumed, but not demonstrated an equivalence between the component of variance due to parent-of-origin effects and that due to imprinting defined by classical quantitative genetics (Santure and Spencer 2006; Campos et al. 2015). Under real-world conditions, a large POE variance component might best be interpreted as identifying a genome-wide pattern of excessive variance among heterozygotes for the phenotype in question which may or may not be due to genomic imprinting.

### Statistical methods

#### Assumptions

Using REML to estimate the variance components model given in Eq. 2 requires that several assumptions (both statistical and genetic) be met in order for inference about the model to be legitimate. In addition to the normality assumptions given above, *G-REMLadp* fitting of the model in Eq. 3 assumes Hardy–Weinberg Equilibrium (HWE) and accuracy of phasing. Departures from HWE [such as non-random mating and differential allele frequencies in male and female parents (Falconer and Mackay 1996)] break the orthogonality of the POE-coded genotype with the additive-coded genotype and the dominance-coded genotype. Non-random mating could decrease the frequency of heterozygotes, while sex-specific allele frequencies cause the frequencies of the ${a_{mo}}{A_{fa}}$ and ${A_{mo}}{a_{fa}}$ heterozygotes to differ. Accurate phasing is required so that the different heterozygotes are correctly called; if they are not, ${Z_\gamma }$ is measured with error, diluting estimates of the ${Z_\gamma },\,y$ association, hence downward-biasing estimates of $\sigma _{\gamma }^{2}$.

#### Power analysis via Haseman–Elston regression approximation

The power of G-REML analysis can be approximated in a Haseman–Elston (HE) regression framework where each distinct pair of individuals in the sample is used as the unit of analysis (Elston et al. 2000; Chen 2014; Visscher et al. 2014). In this framework, the outcome variable is the centered cross-product of phenotypes for each pair of unrelated individuals in the analysis (denoted ${Y_{ij}}$ for individuals $i$ and $j$) and the predictor variables are the GRM entries under additive-coding (${A_{ij}}$), dominance-coding (${\Delta _{ij}}$), and POE-coding (${\Gamma _{ij}}$) of the pair. The coefficients of the predictors in the associated ordinary least squares regression are estimates of the phenotypic variance due to each type of effect. This is similar to unweighted least squares estimation in covariance structure modelling (Browne 1982). Wald tests of significance for the variance components can be made using the assumption that the residuals in the regression are approximately normally distributed (Chen 2014).

We focus on univariate HE regression. The justification for this is that the standardized coded genotypes are orthogonal at each locus and the population values of all cross-locus, cross-coding correlations are 0 (assuming random mating under a polygenic model where causal loci are randomly distributed along the genome, see the Appendix in the Supplementary Materials for derivations). This means that the products of the GRMs ${\mathbf{A}}$, ${\mathbf{\Delta }}$, and ${\mathbf{\Gamma }}$ are expected to be 0 and that sample cross-locus, cross-coding correlations will tend to decrease with increasing sample size and number of effective loci. Thus, the genetic relationship between two individuals should be entirely captured by their entries in the three GRMs. For example, the correlation between individual *i*’s additive coded genotypes (the $1 \times m$ row vector ${\mathbf{z}}{'_{{\mathbf{\alpha }},{\text{i}}}}$) and individual *v*’s dominance coded genotypes (the $m \times 1$ column vector ${{\mathbf{z}}_{{\mathbf{\delta }},~{\text{v}}}}$) is expected to be 0 and to be bound more closely to 0 with increasing sample size *n*, per Eq. 4. The correlation between codings will have mean 0 and variance ${n^{ - 1}}$ over repeated sampling, which we denote using the ${O_p}$ notation to imply that its value is bounded in probability by Chebychev’s inequality as $n$ increases (Bishop et al. 1975, chap. 14); the equality is approximate because the sample correlation is not strictly uncorrelated with *i* and *v*’s coded genotypes.

4 $$\begin{aligned} {{{\left\{ {{\mathbf{A\Delta }}'} \right\}}_{iv}}}&={m^{ - 2}}{\mathbf{z}}{'_{{\mathbf{\alpha }},{\text{i}}}}{{\mathbf{Z}}_{\mathbf{\alpha }}}'{{\mathbf{Z}}_{\mathbf{\delta }}}{{\mathbf{z}}_{{\mathbf{\delta }},~{\text{v}}}} \\ &={m^{ - 2}}\mathop \sum \limits_{{{\text{all~loci~}}k,~l}} {z_{\alpha ,\,ik}}{{\left\{ {{{\mathbf{Z}}_{\mathbf{\alpha }}}'{{\mathbf{Z}}_{\mathbf{\delta }}}} \right\}}_{kl}}~{z_{\delta ,\,vl}},~{\text{where~}}{{\left\{ {{{\mathbf{Z}}_{\mathbf{\alpha }}}'{{\mathbf{Z}}_{\mathbf{\delta }}}} \right\}}_{kl}}~{\text{is~the~sample~~}} \\ &\quad\quad{{\text{correlation~of}}~{\text{additive~coding~at~locus~}}k,{\text{~dominance~coding~at~locus~}}l} \\ &={m^{ - 2}}\mathop \sum \limits_{{{\text{all~loci~}}k,~l}} {z_{\alpha ,\,ik}}{O_p}\left( {{n^{ - 1}}} \right)~{z_{\delta ,\,vl}} \\ &\approx {m^{ - 2}}{O_p}\left( {{m^2}{n^{ - 1}}} \right) \\ & \approx {O_p}({n^{ - 1}}) \end{aligned}$$

This prediction was supported by the empirical analysis, in which the off-diagonal elements of the GRMs were uncorrelated (${r_{\alpha ,\,\gamma }}=5 \times {10^{ - 5}}$,${r_{\alpha ,\,\delta }}= - 2 \times {10^{ - 4}}$, ${r_{\gamma ,\,\delta }}=6 \times {10^{ - 4}}$). Supplementary Figure S1 illustrates this lack of correlation; while Supplementary Table SI shows that the diagonal elements of the empirical GRMs were close to 1.

Orthogonality between codings means that simple HE regressions of Y on each coded genotype’s GRM will yield the same estimates (and associated Wald test statistics) as a multiple regression, and we can analyze the power of simple regressions but fit multiple components in practice. The Wald test statistic of a variance component, POE for example, is given by ${\chi ^2} \approx F=\hat {\beta }_{\gamma }^{2}/{\text{Var}}\left( {{{\hat {\beta }}_\gamma }} \right)=\hat {\sigma }_{\gamma }^{4}/{\text{Var}}\left( {\hat {\sigma }_{\gamma }^{2}} \right)$. By assuming that a given variance component (i.e. the HE slope) is nonzero, the power of this Wald test depends on the noncentrality parameter of the associated statistic. The derivation of the noncentrality parameter is parallel for each type of coded genotype, so we focus on HE regression of POEs, following the example given for additive variance components by Visscher et al. (2014).

Starting with standardized outcomes and predictor [where *vech* is the operator which transforms a symmetric matrix to a column vector of its lower-diagonal elements (Henderson and Searle 1979)]. Simplifying assumptions are required: (1) $Y$ is approximately normal (very unlikely for a cross-product phenotype) so that the Wald test statistic has an $F$ distribution with 1 degree of freedom in the numerator and $\frac{1}{2}~n\left( {n - 1} \right) - 2$ degrees of freedom in the denominator; and (2) the amount of variance in $Y$ explained by $W$ is so small and the sample size so large that the Wald $F$ test statistic is well approximated by a $\chi _{1}^{2}$ random variable.

We derive results for a set of $m$ independent loci. The numerator of the Wald test statistic is the square of the estimated regression coefficient $\hat {\beta }_{\gamma }^{2}={\text{Cov}}{\left( {{Y_{ij}},\,{\Gamma _{ij}}} \right)^2}/{\text{Var}}{\left( {{\Gamma _{ij}}} \right)^2}.$ For individuals $i$ and $j$, the expected covariance between $Y$ and $\Gamma$ is

5 $$\begin{aligned} Cov\left( {Y_{{ij}} \Gamma _{{ij}} } \right) & = E_{{Genotype}} E_{\varepsilon } E_{{\beta _{\alpha } ,\beta _{\gamma } ,\beta _{\delta } }} \left( {\Gamma _{{ij}} y_{i} y_{j} } \right) \\ \; = & \;E_{{Genotype}} \left\{ {\Gamma _{{ij}} \left( {\mathop \sum \limits_{{\theta \in \left( {\alpha ,\,\Delta ,\,\gamma } \right)}} \mathop \sum \limits_{{\vartheta \in \left( {\alpha ,\,\Delta ,\,\gamma } \right)}} \beta _{\theta } 'z_{{\theta ,\,i}} \beta _{\vartheta } 'z_{{\vartheta ,\,j}} + E_{\varepsilon } \left( {C_{1} \varepsilon _{i} + C_{2} \varepsilon _{j} + C_{3} \varepsilon _{i} \varepsilon _{j} } \right)} \right)} \right\} \\ = & E_{{Genotype}} \left\{ {\Gamma _{{ij}} \left( {\mathop \sum \limits_{{\theta \in \left( {\alpha ,\,\Delta ,\,\gamma } \right)}} \mathop \sum \limits_{{\vartheta \in \left( {\alpha ,\,\Delta ,\,\gamma } \right)}} \beta _{\theta } 'z_{{\theta ,\,i}} \beta _{\vartheta } 'z_{{\vartheta ,\,j}} + E_{\varepsilon } \left( {C_{1} \varepsilon _{i} + C_{2} \varepsilon _{j} + C_{3} \varepsilon _{i} \varepsilon _{j} } \right)} \right)} \right\} \\ = & E_{{Genotype}} \left\{ {\Gamma _{{ij}} \left( {\mathop \sum \limits_{{\theta \in \left( {\alpha ,\,\Delta ,\,\gamma } \right)}} \mathop \sum \limits_{{\vartheta \in \left( {\alpha ,\,\Delta ,\,\gamma } \right)}} \mathop \sum \limits_{k}^{m} \mathop \sum \limits_{l}^{m} \beta _{{\theta ,\,l}} \beta _{{\vartheta ,\,k}} z_{{\theta ,\,il}} z_{{\vartheta ,\,jk}} } \right)} \right\} \\ = & E_{{Genotype}} \left\{ {\Gamma _{{ij}} \left( {\mathop \sum \limits_{\vartheta } \mathop \sum \limits_{k}^{m} \beta _{{\vartheta ,\,k}}^{2} z_{{\theta ,\,ik}} z_{{\vartheta ,\,jk}} + \mathop \sum \limits_{{\theta \ne \vartheta }} \mathop \sum \limits_{{k \ne l}} \beta _{{\theta ,\,l}} \beta _{{\vartheta ,\,k}} z_{{\theta ,\,il}} z_{{\vartheta ,\,jk}} } \right)} \right\} \\ = & E_{{Genotype}} \left\{ {\Gamma _{{ij}} \left( {\sigma _{\alpha }^{2} A_{{ij}} + \sigma _{\Delta }^{2} \Delta _{{ij}} + \sigma _{\gamma }^{2} \Gamma _{{ij}} + O_{p} \left( {n^{{ - 1}} } \right)} \right)} \right\} \\ = & \sigma _{\gamma }^{2} E\left( {\Gamma _{{ij}}^{2} } \right) = \sigma _{\gamma }^{2} Var\left( {\Gamma _{{ij}} } \right) \\ \end{aligned}$$

recalling that the coded genotypes are orthogonal within a locus and using the expectation of vanishing cross-locus, cross-coding correlations given in the Supplementary Material. This derivation shows that the HE regression coefficient in the population is $\sigma _{\gamma }^{2}$, hence the numerator of the Wald test statistic is $\sigma _{\gamma }^{4}$.

The denominator of the Wald $F$ test statistic is the error variance per degree of freedom, divided by the variance of the predictor variable, i.e. ${\text{Var}}\left( \epsilon \right)/\left( {{\text{Var}}\left( {{\Gamma _{ij}} \times df} \right)} \right)$. Here we use the approximation that ${\text{Var}}\left( \epsilon \right) \approx {\text{Var}}\left( Y \right)=1$. HE regression uses the distinct pairs of observations as the units of analysis, so the denominator degrees of freedom are $\frac{1}{2}~n\left( {n - 1} \right)$ when estimating the variance component and an intercept term. The variance of the POE GRM calculated at any single locus can be shown by direct calculation to be 1, which is also true for the additive component and dominance component GRMs (given assumptions). ${\text{Var}}\left( {{\Gamma _{ij}}} \right)$ is interpretable as the variance of the average of these component GRMs over ${m_e}$ loci, hence is $m_{e}^{{ - 1}}$ in this simple model. A more accurate approach (for additive variance components) is given in Appendix 1 of Goddard (Goddard 2009), in which the variance in relatedness is averaged over the number of effective loci given variation in pedigree as well as linkage disequilibrium. Visscher et al. give an empirical estimate of the number of effective loci for GRMs calculated using genome-wide common SNPs in the HapMap3 panel, hence they suggest using ${\text{Var}}\left( {{A_{ij}}} \right)=2 \times {10^{ - 5}}$ (Visscher et al. 2014). In our model, the denominator of the Wald test statistic is $1/\left( {m_{e}^{{ - 1}}\left( {0.5n\left( {n - 1} \right) - 2} \right)} \right) \approx 2{m_e}/{n^2}$. This means the mean Wald test statistic is approximately ${n^2}\sigma _{\gamma }^{4}/2{m_e}$, which is referred to a $\chi _{1}^{2}$ distribution because the denominator degrees of freedom in the $F$-test are very large. This derivation is nearly identical to that used in (Visscher et al. 2014), so it is possible to use their online tool (http://cnsgenomics.com/shiny/gctaPower/) to determine the power to detect a variance component of a particular size.

For ${m_e}$ loci and sample size $n$, the noncentrality parameters of the $\chi _{1}^{2}$ test statistic are:

6 $$\begin{array}{*{20}{r}} {{\lambda _\alpha }}&{=0.5m_{e}^{{ - 1}}n\left( {n - 1} \right)\sigma _{\alpha }^{4}} \\ {{\lambda _\delta }}&{=0.5m_{e}^{{ - 1}}n\left( {n - 1} \right)\sigma _{\delta }^{4}} \\ {{\lambda _\gamma }}&{=0.5m_{e}^{{ - 1}}n\left( {n - 1} \right)\sigma _{\gamma }^{4}} \end{array}$$

In our simulations, we used the true $m$, as loci were simulated without LD; in practice, we recommend the approximation $m_{e}^{{ - 1}} \approx 2 \times {10^{ - 5}}$ (Visscher et al. 2014), although its application to dominance variance components and POE-based variance components is based only on analogy with additive variance components and the number of effective loci will differ depending on which SNP panel is used.

### Implementation

*G-REMLadp* requires a set of phased genotypes, each with parent-of-origin assignments. Mitochondrial DNA and X chromosome SNPs are excluded from analysis. In the empirical analysis, we used a *Perl* script to assign parent-of-origin to genotypes which had already been phased, as described below. Given a set of phased, parental-origin-assigned SNPs, we first recoded each genotype to the three-term orthogonal coding given in Table 1, stored this data in the software package *MACH*’s (Li et al. 2010) “dosage” format, and then called *GCTA* to generate GRMs for the three variance components. *GCTA*’s—make-grm and—make-grmd (Zhu et al. 2015) make the appropriate GRMs for the additive and dominance components directly from the *MACH*-formatted phased genotype. However, for POEs, we recoded each locus (in *R*) by subtracting the paternal minor allele indicator from the maternal indicator and wrote this to an appropriately formatted .mldose.gz file, then generated an .mlinfo.gz file from the relevant sources for the SNP data set. We input the .mldose.gz and .mlinfo.gz files to *GCTA*’s MACH dosage function (--dosage-mach-gz) to generate the POE GRM. We then input the additive, dominance, and POE GRMs to *GCTA*, with the phenotype and covariate files, to fit the mixed model and estimate variance components. We chose to estimate the variance components without constraining them to be positive so that the null distribution of test statistics would not be a mixture (Visscher 2006). We also used the AI-REML algorithm instead of the Newton–Raphson or EM algorithms to fit the model quickly. Scripts to run these analyses (as well as to perform the simulations described below) are available at the GitHub repository (https://github.com/amatrhr/g-remladp); power calculations can be performed using the GCTA-GREML power calculator at (http://cnsgenomics.com/shiny/gctaPower/).

### Simulations

We simulated data according to the *G-REMLadp* model to: (1) evaluate the predicted statistical power using the Haseman–Elston regression approximation; (2) test the bias and variance of the method in response to violation of its assumptions; and (3) assess computational requirements. In simulation studies, data were simulated according to Eq. 1, and variance components were estimated using both HE regression and *GCTA* software. The goals were to estimate the bias and variance of the variance component estimators, the agreement between HE and *GCTA* estimates, and the empirical power and Type I error rates of the HE test.

The design factors were: sample size $n=1000,\,2000,\,4000,\,5000,\,7500$, $m=500,\,1000,\,3000,\,5000$ SNPs; $\sigma _{\alpha }^{2}/{\text{Var}}\left( y \right)=0,\,0.017,\,0.033,\,0.1$; $\sigma _{\delta }^{2}/{\text{Var}}\left( y \right)=0,\,0.017,\,0.033,\,0.1$; $\sigma _{\gamma }^{2}/{\text{Var}}\left( y \right)=0,\,0.017,\,0.033,\,0.1$; a violation of HWE—simulating maternal and paternal genotypes with different minor allele frequencies (either no difference, or mothers’ allele frequency greater than fathers’ by 0.05, meaning that the HWE test statistic will have a noncentrality parameter of > 100 and power over 90% for mothers’ MAF > 0.10 and $n$ > 1000) so that the POE-coded genotype is no longer orthogonal to the other two codings (which remain mutually orthogonal); and a second violation of HWE-simulating parental genotypes to be correlated at $r=0$ or $r=0.25$, on average across all simulated loci, so that the diagonal entries of the GRMs are on average less than 1 and results might be less stable numerically. The sample size and number of effective loci used were constrained to be small due to computational requirements; computing GRMs at genome scale ($n>5000,\,{m_e}>{10^6}$) and fitting models to them required 100% use of at least 16 processors for approximately an hour, which is feasible for single empirical analyses but not in factorial simulations with thousands of replications. The SNPs were simulated to be independent with minor allele frequencies uniformly distributed between 0.01 and 0.5. Because there were relatively few of them, the simulated SNPs had much larger individual effects than would be expected in empirical data. We chose to simulate violations of HWE to test the robustness of *G-REMLadp* to violations of its assumptions, and as a way of widening the breadth of the simulations. Realistically, most samples in which *G-REMLadp* could be applied will have filtered SNPs for violations of HWE as part of routine QC (Laurie et al. 2010). A total of 7500 replications were simulated, but in the simulation results presented here, these factors were not completely crossed.

Three measures were used to evaluate the method: (1) empirical absolute bias, the average difference between the simulated variance component and the value estimated in a replication; (2) empirical sampling variance, the variance of estimated variance components over replications; and (3) power/Type I error rate, the proportion of replications in which the Wald test of a null variance component exceeded the 95% critical value under a simulated non-null/null variance component.

In the simulations, we expected power to increase with increasing sample size and variance component size and decreasing number of effective loci (noting that this means a larger effect at each locus for a given variance component size in our design), and expected variance to decrease according to the same pattern. We expected that violation of assumptions would lead to detectable bias.

### Empirical analysis

We applied *G-REMLadp* to a sample of up to 4753 individuals gathered as part of the Avon Longitudinal Study of Parents and Children (ALSPAC), a prospective study of health and development beginning at pregnancy. We considered 36 different phenotypes which had been previously associated with POEs or which were related to body size, development, or social functioning.See the Supplementary Material for further description of the sample, including the phenotyping and quality control procedures that we applied to it. The sample, the longitudinal study and its context, as well as its genotyping, are described in detail in two papers (Boyd et al. 2012; Fraser et al. 2013). The 36 phenotypes are listed, with summary statistics, in Supplementary Table SII. G-REMLadp estimates of additive variance components, dominance variance components, and POE variance, are listed in Supplementary Table SIII. Highly skewed phenotypes (listed in Supplementary Methods) were inverse-normal transformed prior to estimating variance components (Peng et al. 2007); fixed effects of sex and the first four ancestry-informative principal components were modelled. Parental effects and POEs have been previously studied in trios in this sample (Davey Smith et al. 2007), but not genome-wide for the 36 phenotypes.

We also used the ALSPAC data to test our predictions that variance components estimated simultaneously using G-REMLadp would agree with: variance components estimated in a univariate fashion using G-REMLadp, and also with HE regression estimates. The agreement among these methods was substantial ($r>0.98$ in all cases); univariate G-REMLadp results are givien in Supplementary Table SIV; univariate HE regression estimates are given in Supplementary Table SV.

## Results

### Simulation results

#### Power and sample size

**Fig. 2:**
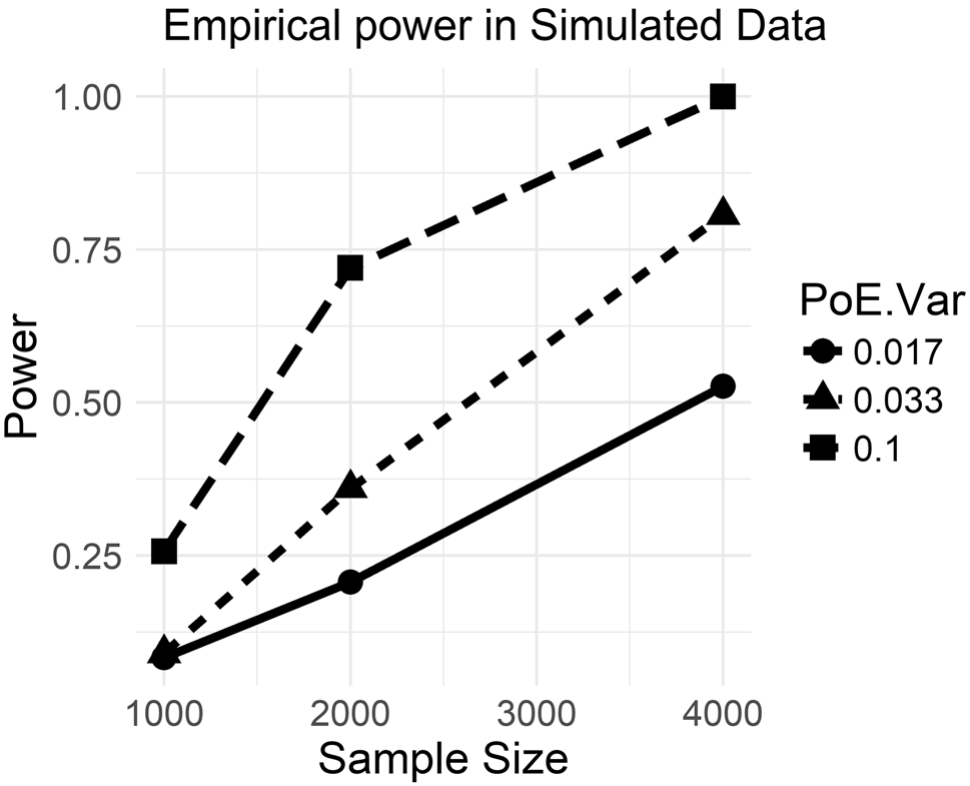
Empirical power curves in simulated data: Curves generated using proportions of significant Wald tests in simulated data with all assumptions specified in the text satisfied. %PoE: proportion of phenotypic variance attributable to parent-of-origin effects in the model used to generate simulated data; Sample size: number of simulated individuals with parent-of-origin determined at all loci; Power: proportion of replicates with Wald tests having $p$-values $<0.05$. Note that the number of effective loci in this figure is an order of magnitude lower than in Supplementary Figure S2

Sample size curves at a Type I error rate of 5%, based on the HE approximation, are given in Supplementary Figure S2, for POEs responsible for 1%, 3%, 10%, and 15% of the total phenotypic variance explained, tagged by 10,000 loci. Supplementary figure S2 shows that a sample size of over 10,000 genotyped duos or trios, with probands having phased genotypes, is likely necessary to detect the largest conceivable parent-of-origin effect variances and that a sample of 50,000 individuals will be needed to detect POEs accounting for $\approx 1 - 3\%$ of phenotypic variance, which was the size observed by Lopes et al. (2015). Figure 2 gives the empirical power, which was high because such large variance components were simulated and because ${m_e}$ was low, with a median value of 2000 effective loci.

The test involving HE regression is based on further simplifying and adding assumptions to the *G-REMLadp* procedure, hence discrepancies between the two approaches were expected, with the concern that large discrepancies would render the approximate power calculations unhelpful. Table SVI, in the Supplementary Material, shows that the simulated HE-based test statistics lay relatively close to their expected values. Supplementary Figures S3–S5 illustrate this graphically.

#### Type I error rates

There was no evidence that the tests of the POEs had inflated Type I error rates when assumptions were met; under these conditions, the simulated type I error rate was 0.0510. The HE-based test of the POE variance component had slightly elevated Type I error rates (0.0625) under violation of HWE due to dependence of parental genotypes.

#### Bias and variance due to violating assumptions

We observed that violating HWE increased the discrepancy between predicted and observed test statistics. However, these results do not indicate whether this was due to increased bias or variance of the estimates, or both. Additionally, because the predicted test statistics were based on many simplifying assumptions, it is possible that the increased discrepancy would not have led to incorrect inference, and it is worth exploring whether violating HWE causes the estimated variance components to be misleading in predictable ways.

Results for simulations with truly null variance components are not shown. The absolute bias was under $3 \times {10^{ - 3}}$ for each type of variance component under all simulation conditions. Variances were similar to those for non-null estimates.

Table 2 shows the GCTA results under HWE. The bias was small, less than 5% of the true parameter value for each type of variance component. There may be a tendency to increased bias and variance at larger effect sizes. Results were similar for HE model fitting.

**Table 2:** Absolute bias and variance for *G-REMLadp* variance components when HWE was met, for non-null simulated effects

N	$\sigma _{\alpha }^{2}$	$\sigma _{\delta }^{2}$	$\sigma _{\gamma }^{2}$	#Reps	$\hat {\sigma }_{\alpha }^{2}$ Bias	$\hat {\sigma }_{\alpha }^{2}$ Var	$\hat {\sigma }_{\delta }^{2}$ Bias	$\hat {\sigma }_{\delta }^{2}$ Var	$\hat {\sigma }_{\gamma }^{2}$ Bias	$\hat {\sigma }_{\gamma }^{2}$ Var
1000	0.017	0.017	0.017	280	1.0e−03	1.1e−03	1.5e−03	8.9e−04	1.2e−03	1.1e−03
1000	0.033	0.033	0.033	290	− 1.1e−04	1.9e−03	− 1.7e−03	1.8e−03	− 2.0e−03	1.8e−03
1000	0.100	0.100	0.100	290	3.0e−03	7.1e−03	9.0e−04	5.7e−03	2.6e−03	6.3e−03
2000	0.017	0.017	0.017	300	− 2.2e−03	2.9e−04	− 6.5e−05	2.6e−04	4.2e−04	2.6e−04
2000	0.033	0.033	0.033	300	− 2.7e−04	5.7e−04	− 1.9e−03	4.9e−04	1.2e−03	6.3e−04
2000	0.100	0.100	0.100	300	3.7e−03	1.6e−03	1.6e−03	1.7e−03	−1.1e− 03	1.6e−03
4000	0.017	0.017	0.017	300	5.0e−04	6.5e−05	1.2e−05	6.8e−05	−8.0e−04	7.5e−05
4000	0.033	0.033	0.033	300	2.9e−05	1.7e−04	−6.2e−04	1.5e−04	− 8.0e−04	1.4e−04
4000	0.100	0.100	0.100	300	− 2.6e−03	4.8e−04	− 1.8e−03	4.4e−04	1.5e−03	4.5e−04

Proportion of phenotypic variance explained: by additive effects given in the $\sigma _{\alpha }^{2}$ column, by dominance effects given in the $\sigma _{\delta }^{2}$ column, and by parent-of-origin effects given in the $\sigma _{\gamma }^{2}$ column. N: number of simulated phased genotypes, #Reps: number of simulated replications, “Bias” is the absolute bias of estimates across simulated replications, “Var” is variance of estimates across simulated replications

Table 3 shows the GCTA results when the HWE assumptions were violated. Additive variance components estimates did not seem to be biased or to have increased variance under violation of HWE. When parental gametes were correlated, parent-of-origin effect variance estimates were downward biased, sometimes as much as 10 or 20% of the parameter value. Biases due to correlated gametes in estimating dominance variance components were smaller but were uniformly positive. Variance of estimates did not seem to be affected by violation of HWE. Results were similar for HE model fitting.

**Table 3:** Absolute bias and variance for *G-REMLadp* variance components when HWE was violated, for non-null simulated effects

Parent Corr	MAF Diff	N	$\sigma _{\alpha }^{2}$	$\sigma _{\delta }^{2}$	$\sigma _{\gamma }^{2}$	#Reps	$\hat {\sigma }_{\alpha }^{2}$ Bias	$\hat {\sigma }_{\alpha }^{2}$ Var	$\hat {\sigma }_{\delta }^{2}$ Bias	$\hat {\sigma }_{\delta }^{2}$ Var	$\hat {\sigma }_{\gamma }^{2}$ Bias	$\hat {\sigma }_{\gamma }^{2}$ Var
0.00	0.05	1000	0.017	0.017	0.017	300	1.7e−03	1.0e−03	− 4.8e−04	9.7e−04	2.9e−03	1.0e−03
0.00	0.05	1000	0.033	0.033	0.033	300	1.1e−03	2.4e−03	1.1e−03	2.4e−03	6.6e−04	2.0e−03
0.00	0.05	1000	0.100	0.100	0.100	300	− 1.0e−03	6.6e−03	2.9e−04	6.4e−03	3.1e−04	5.7e−03
0.00	0.05	2000	0.017	0.017	0.017	300	− 1.4e−03	2.5e−04	3.5e−04	3.1e−04	− 1.8e−05	2.8e−04
0.00	0.05	2000	0.033	0.033	0.033	300	9.4e−04	5.4e−04	1.6e−03	5.2e−04	3.0e−04	4.8e−04
0.00	0.05	2000	0.100	0.100	0.100	300	2.2e−03	1.6e−03	− 5.2e−04	1.6e−03	6.9e−04	1.6e−03
0.00	0.05	4000	0.017	0.017	0.017	300	1.9e−04	7.2e−05	− 6.7e−04	6.9e−05	− 9.4e−04	8.1e−05
0.00	0.05	4000	0.033	0.033	0.033	300	− 6.3e−04	1.5e−04	8.3e−04	1.8e−04	− 8.2e−04	1.5e−04
0.00	0.05	4000	0.100	0.100	0.100	290	− 2.1e−03	4.9e−04	1.7e−03	5.6e−04	− 2.0e−04	4.3e−04
0.25	0.00	1000	0.017	0.017	0.017	300	6.1e−04	8.2e−04	1.5e−03	1.6e−04	− 1.4e−03	1.1e−03
0.25	0.00	1000	0.033	0.033	0.033	290	1.9e−03	1.6e−03	1.9e−03	2.5e−04	− 6.8e−03	2.0e−03
0.25	0.00	1000	0.100	0.100	0.100	290	1.7e−03	5.6e−03	2.1e−03	9.8e−04	− 2.5e−02	8.3e−03
0.25	0.00	2000	0.017	0.017	0.017	300	− 1.3e−04	2.0e−04	− 3.5e−04	5.6e−05	− 5.4e−03	2.8e−04
0.25	0.00	2000	0.033	0.033	0.033	300	2.0e−03	3.9e−04	− 9.0e−05	1.0e−04	− 6.9e−03	6.5e−04
0.25	0.00	2000	0.100	0.100	0.100	300	3.6e−03	1.5e−03	4.5e−03	3.8e−04	− 2.5e−02	2.2e−03
0.25	0.00	4000	0.033	0.033	0.033	300	2.3e−04	1.2e−04	4.0e−04	4.6e−05	− 7.1e−03	1.3e−04
0.25	0.00	4000	0.100	0.100	0.100	300	1.3e−03	3.6e−04	1.7e−03	1.4e−04	− 2.3e−02	4.7e−04
0.25	0.05	1000	0.017	0.017	0.017	130	− 4.1e−03	8.4e−04	3.5e−03	3.6e−04	− 3.4e−03	1.3e−03
0.25	0.05	1000	0.033	0.033	0.033	160	− 1.2e−03	1.7e−03	2.1e−04	5.6e−04	− 1.5e−02	2.5e−03
0.25	0.05	1000	0.100	0.100	0.100	220	− 9.1e−04	5.2e−03	7.1e−03	2.6e−03	− 3.1e−02	8.2e−03
0.25	0.05	2000	0.017	0.017	0.017	280	− 9.5e−04	1.9e−04	4.4e−05	1.1e−04	− 4.7e−03	3.2e−04
0.25	0.05	2000	0.033	0.033	0.033	300	8.4e−05	3.8e−04	2.0e−03	2.8e−04	− 8.6e−03	5.4e−04
0.25	0.05	2000	0.100	0.100	0.100	300	5.2e−03	1.4e−03	1.2e−03	7.4e−04	− 2.1e−02	1.8e−03
0.25	0.05	4000	0.033	0.033	0.033	300	− 2.9e−04	1.3e−04	1.5e−03	7.8e−05	− 7.8e−03	1.6e−04
0.25	0.05	4000	0.100	0.100	0.100	300	2.2e−03	3.1e−04	3.5e−03	2.5e−04	− 2.3e−02	4.8e−04

*Parent Corr* average correlation of parental genotypes, *MAF Diff* average difference in parental MAFs. Proportion of phenotypic variance explained by additive effects is given in the $\sigma _{\alpha }^{2}$ column, by dominance effects in the $\sigma _{\delta }^{2}$ column, and by parent-of-origin effects in the $\sigma _{\gamma }^{2}$ column. *N* number of simulated phased genotypes, *#Reps* number of simulated replications, “Bias” is absolute bias of estimates across simulated replications, “Var” is variance of estimates across simulated replications

The *GCTA* and HE estimates tended to differ in the presence of correlation between parental genotypes; in this situation, HE regression estimates of POEs had higher variance (and hence higher mean-squared error) than did *GCTA* estimates, which is expected to be the case even in additive-only models (Yang et al. 2017). As a result, the correspondence between the two methods decreased from a HE-*GCTA* correlation of 0.9911(0.9908, 0.9915) with independent parental genotypes to 0.9624(0.9597, 0.9649) with correlated parents. This was not observed for estimates of the two other types of variance components.

### Empirical analysis

**Fig. 3:**
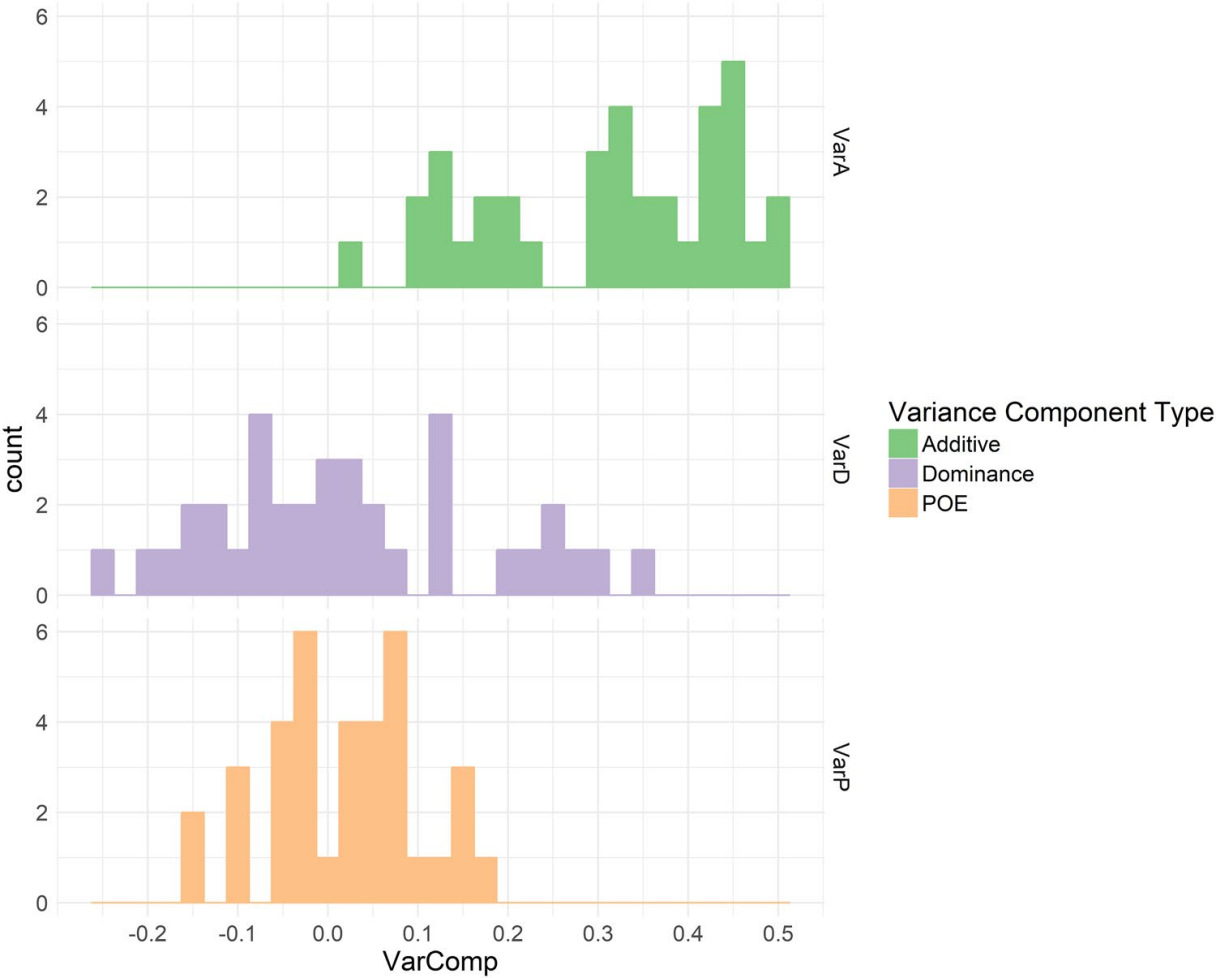
Histograms of the estimates of additive, dominance, and parent-of-origin variance components for 36 ALSPAC traits. *Add* Estimated proportions of phenotypic variance attributable to additive effects, *Dom* Estimated proportions of phenotypic variance attributable to dominance effects, *PoE* Estimated proportions of phenotypic variance attributable to parent-of-origin effects. The bin width is 0.025. To avoid biased variance estimates, they were not constrained to be positive; hence, for POEs, which the study was underpowered to detect, there are many negative estimates. The figure is intended to illustrate patterns in the variance component estimates for the phenotypes which happened to be included in this study; it would be inappropriate to consider these histograms as representative of the densities of different variance components for complex traits in general

Estimates and standard errors of $\sigma _{\alpha }^{2}$, $\sigma _{\delta }^{2}$, and $\sigma _{\gamma }^{2}$, calculated using GCTA to fit a multiple-component *G-REMLadp* are given in Supplementary Table III for the 36 phenotypes. Results for a set of five variables with large parent of origin variance component estimates are given in Table 4. The sample size of $n \approx 5000$ was sufficient to detect established additive variance components for anthropometric variables such as height and fat mass. The mean estimate for additive variance components was 0.310(0.14). There is evidence for dominance variance on verbal IQ and fat mass in the sample. The mean estimate for dominance variance components was 0.026(0.15). The largest (and most reliably estimated) POE variance components were estimated for age at menarche, FVC, age at first tooth, and blood pressure. The mean estimate for POE variance was 0.018(0.08). Interestingly, height had a large, negative estimate of the POE variance component. Figure 3 presents histograms of the variance component estimates, using different colors to represent additive vs dominance vs POE variance component estimates in the 36 ALSPAC traits. Figure 3 is broadly similar to Fig. 2 of (Zhu et al. 2015), which contains histograms of variance component estimates for additive and dominance variance component estimates in 79 traits from the Atherosclerosis Risk in Communities cohort.

**Table 4:** Large *G-REMLadp* parent of origin variance component estimates for five ALSPAC phenotypes

Phenotype	VarA	SEvarA	VarD	SEvarD	VarP	SEvarP
Age at first tooth	0.425	0.083	0.114	0.121	0.156	0.082
Age at Menarche	0.337	0.162	0.195	0.246	0.179	0.162
DBP	0.193	0.088	− 0.076	0.132	0.114	0.088
FVC	0.370	0.097	0.017	0.136	0.159	0.097
SBP	0.313	0.088	− 0.147	0.130	0.145	0.088

Variance component estimates of five standardized phenotypes in ALSPAC, estimates and standard errors generated with multiple components GCTA-GREML. *VarA* proportion of phenotypic variance attributable to additive genetic effects, *SEvarA* standard error of proportion of phenotypic variance attributable to additive genetic effects. *VarD* proportion of phenotypic variance attributable to dominance effects, *SEvarD* standard error of proportion of phenotypic variance attributable to dominance effects. *VarP* proportion of phenotypic variance attributable to parent-of-origin effects, *SEvarP* standard error of proportion of phenotypic variance attributable to parent-of-origin effects. *DBP* diastolic blood pressure. *FVC* forced vital capacity. *SBP* systolic blood pressure. See Supplementary Tables SIII for results on all phenotypes and SVII for unstandardized variance components

Across the 36 phenotypes surveyed, in the aggregate, additive variance components were frequently estimated reliably, positively, and away from 0. In general, if a variance component was detectable, it represented additive variance. POEs had standard errors approximately equal to those of additive variance components, while estimates of dominance variance showed slightly larger standard errors. Further, half of the dominance variance components were negative and half positive. Although the sample size is too small to perform significance tests of differences in heritability estimates across traits, lipid traits tended to have negative dominance components and IQ traits positive ones.

## Discussion

The most important findings from our simulations are that: (1) *G-REMLadp* does not seem to be inherently biased in estimating variance due to additive effects, dominance effects, and POEs, and (2) that substantial correlation between parental genotypes is necessary to bias *G-REMLadp* estimates. We did not investigate the effect that linkage disequilibrium might have on our results, but present the correlations between codings in the Supplementary Material. If local levels of linkage disequilibrium are associated with POE size, an implementation of *G-REMLadp* using GCTA-LDMS would likely be able to estimate variance components without LD bias, while an LDAK implementation would have estimates at greater risk of bias (Speed et al. 2012; Yang et al. 2017). The empirical results and power calculations suggest that a sample size under 10,000 is insufficient to generate precise *G-REMLadp* estimates. Hence, POEs of the size observed by Lopes et al. (Lopes et al. 2015) ($\approx 2\%$ of variance explained) are likely to require sample sizes close to 50,000 to resolve properly (their samples were about 4500 purebred pigs, hence ${m_e}$ far below that for humans on the HapMap 3 panel, accordingly their pattern of results is closer to our simulated findings than to our empirical ones). It is unlikely that power could be improved substantially via improved phasing using trio samples due to the already high accuracy of phasing using parent–child duos in the case of dense genome-wide SNP data (Marchini et al. 2006; Browning and Browning 2011).

Despite the limited power of our analysis, it may be interesting to follow up some of the phenotypes that had high estimates of parent of origin effects in the ALSPAC cohort, like age at menarche [a phenotype known to be influenced by imprinted loci (Perry et al. 2014)] in a larger study. Replicating the dominance results for fat mass would also be of interest, as Zhu et al. (2015) found no significant dominance for skinfold thickness; similarly, replicating a dominance heritability component of verbal IQ would substantiate venerable claims for non-additive effects on cognitive performance (Devlin et al. 1997; Plomin and Deary 2015).

However, the highest-profile claim (Devlin et al. 1997) for non-additive effects on IQ involves maternal effects. The evidence for this claim comes from findings of increased similarity in IQ in the children of female twins relative to the children of male twins (Nance and Corey 1976). Maternal (or paternal) effects are defined as indirect effects of the parent’s genotype on offspring phenotype, usually via parental influence on offspring’s environment (Wolf and Wade 2009). These are distinct from the POEs discussed here, because POEs are direct effects of the offspring’s genotype on its phenotype. In the presence of maternal (paternal) effects, the mother’s (father’s) genotype explains some of the residual phenotypic variation remaining after regressing the offspring’s phenotype on its genotype. However, methods designed to detect POEs by modelling different distributions of the two heterozygotes’ phenotypes (including *G-REMLadp*) cannot distinguish POEs from certain patterns of maternal/paternal effects (Hager et al. 2008). It is also possible that there are maternal effects that cause imprinting (epigenetic changes due to intrauterine environment or family environment created by parental behavior). To distinguish POEs from maternal effects, it might be possible to fit a model with both maternal and POEs. For example, *G-REMLadp* could be implemented in OpenMx and combined with the M-GCTA model (Eaves et al. 2014; Kirkpatrick and Neale 2015; Neale et al. 2015). However, POEs and maternal/paternal effects represent departure from Mendelian inheritance; a reliable nonzero $\sigma _{\gamma }^{2}$ value detected by *G-REMLadp* is worthy of follow-up even if it represents a mixed bag of POEs and indirect effects.

Other than the insufficient sample size in the ALSPAC analyses, an additional limitation of this project was that the simulations and empirical data were not closely matched; less idealized, more informative simulations could have been performed. For example, simulated phenotypes could have been based on the empirical distributions of the ALSPAC phenotypes, and these, coupled with real GRMs (with imperfect HWE because of sampling error, if not actual violations), could’ve resulted in more thorough evaluation of *G-REMLadp*. Pairwise relatedness values could also have been simulated from the empirical distributions of (${A_{ij}}$, ${\Delta _{ij}}$, and ${\Gamma _{ij}}$) or smooth approximations to them (Lee and Chow 2014). This approach is more feasible computationally under the HE regression framework than under maximum likelihood. The extreme-seeming violations of HWE which we used (parents’ gametes highly correlated or MAF differences between parents) are observable in certain human populations. Several localities where consanguineous marriages are common are identified in Erzurumluoglu et al. (2016). Examples include the city of Riyadh, in which first-cousin marriages (corresponding to correlation between parental gametes of 0.125) are 30–40% of all marriages; this paper also lists localities where uncle-niece marriages (gametic correlation of 0.25, as we have simulated) represent several percent of all marriages. Erzurumluoglu et al. also describe Riyadh as composed of recently immigrated subpopulations, which makes allele frequency differences possible in the remainder of marriages there.

A further drawback is that the power calculations which we present are based on the assumption that causal loci are randomly distributed throughout the genome. It is unclear the degree to which parent of origin effects are randomly distributed across the genome in humans vs being clustered at distinct loci. Dissecting the genetic basis of parent of origin effects in humans has been hampered by two factors: (1) GWAS aimed at detecting parent of origin effects have been small meaning that only a small number of common SNPs having relatively large effect have so far been identified; and (2) Many studies have restricted association analyses to regions of the genome known to be imprinted. There are anywhere from 40 to 100 regions known to be imprinted in humans (Baran et al. 2015), although recent evidence suggests that this figure may be higher (Cuellar Partida et al. 2017). Additionally, whilst imprinting is one mechanism through which parent of origin effects may manifest, there are other processes (e.g. maternal effects) that may also leave wide-spread signatures across the genome that are largely consistent with the results from parent of origin analyses. Since *G-REMLadp* is not necessarily limited to genome-wide analyses; future work could specifically examine relatedness at known imprinted loci in the construction of GRMs, although this would require revised power calculations to estimate sufficiently large samples to account for the lower degree of tagging of phenotypic variation.

A 2015 paper by Baran et al. in *Genome Research*, supplies an atlas of imprinted regions in the genome (Baran et al. 2015). A related future direction (as suggested by Lopes et al.) would be to emphasize the connection between imprinting and attempts to detect POEs, for example by applying the method in the EWAS, rather than GWAS, context, i.e. identifying genome-wide eQTL effects on age at menarche and first tooth, and 2d4d ratio (Zhu et al. 2016).

In summary, *G-REMLadp* offers the ability to easily partition phenotypic variance according to three types of inherited effects. Given large studies of parent–child trios/duos (for example, MoBa), it is possible to fit *G-REMLadp* models and detect variance components without bias. The close agreement between GREML and HE regression approaches to estimate variance components suggests that *G-REMLadp* models can be fit to a good approximation even in very large studies or with limited computational resources, where HE can be up to 50 times faster than GREML (Yang et al. 2017). Finally, failure to model POEs has been suggested as one possible source of missing heritability (Kong et al. 2009; Eichler et al. 2010). For any phenotypes with missing heritability that is uncovered by modelling POEs, the epigenetic and evolutionary implications of POEs lead to hypotheses of distinctive etiologies and genomic architectures.

## Electronic supplementary material

Below is the link to the electronic supplementary material.

Supplementary material 1 (DOCX 796 KB)
