# Supplementary material for: Partitioning Phenotypic Variance Due to Parent-of-Origin Effects Using Genomic Relatedness Matrices
Source: Behav Genet. 2017 Nov 2;48(1):67–79. doi: 10.1007/s10519-017-9880-0 (PMC5752821; doi:10.1007/s10519-017-9880-0)
Supplement: Supplementary file 1 — Supplementary material 1 (DOCX 796 KB) [file 10519_2017_9880_MOESM1_ESM.docx]

Supplement to Partitioning Phenotypic Variance due to Parent-of-Origin Effects using Genomic Relatedness Matrices

Contents

[Sample Description 2](#_Toc494054821)

[Phenotyping Methods 3](#_Toc494054822)

[Supplementary Tables 10](#_Toc494054823)

[Table SI: Diagonal elements of ALSPAC GRMs 10](#_Toc494054824)

[Table SII: Descriptive Statistics of 36 ALSPAC Phenotypes 12](#_Toc494054825)

[Table SIII: GCTA Estimates of G-REMLadp models in ALSPAC 14](#_Toc494054826)

[Table SIV: Single-component GCTA Estimates of G-REMLadp models in ALSPAC 16](#_Toc494054827)

[Table SV: HE Regression Estimates of G-REMLadp models in ALSPAC 18](#_Toc494054828)

[Table SVI: Accuracy of $\boldsymbol{\chi}\mathbf{2}$ approximations across all simulations 21](#_Toc494054829)

[Table SVII: GCTA Estimates of G-REMLadp models of unstandardized phenotypes in ALSPAC 22](#_Toc494054830)

[Supplementary Figures 25](#_Toc494054831)

[S1: Scatterplot matrix of off diagonal elements of ALSPAC GRMs 25](#_Toc494054832)

[S2: Expected power to detect parent-of-origin effects tagged by $\boldsymbol{m}=\mathbf{10000}$ loci 27](#_Toc494054833)

[S3: Wald test statistics for simulated additive effects 30](#_Toc494054834)

[S4: Wald test statistics for simulated dominance effects 33](#_Toc494054835)

[S5: Wald test statistics for simulated parent-of-origin effects 35](#_Toc494054836)

[Appendix: Correlations between codings of phased genotypes at two loci 37](#_Toc494054837)

[Notation and Parental gametes, considering two linked loci 37](#_Toc494054838)

[Correlation between additive codings 38](#_Toc494054839)

[Correlation between parent-of-origin codings 38](#_Toc494054840)

[Correlation between dominance codings 38](#_Toc494054841)

[Cross-locus, cross-coding correlation: additive and parent-of-origin 39](#_Toc494054842)

[Cross-locus, cross-coding correlation: additive and dominance 40](#_Toc494054843)

[Cross-locus, cross-coding correlation: parent-of-origin and dominance 40](#_Toc494054844)

[References 41](#_Toc494054845)

### Sample Description

ALSPAC is a geographically limited birth cohort based on women in Avon (southwestern England) who were pregnant with due-dates between April and December 1991, inclusive (Boyd *et al.*, 2012; Fraser *et al.*, 2013) ^­^. The initial sample size was 15247 pregnancies, from which 14701 children survived to age 1. Over the next three decades, this set of parents (including mothers' partners) and children has completed an extensive set of questionnaires, clinical studies, and biological samples. Parental consent and child's assent (and child's consent, upon majority) were obtained for each measurement occasion. Ethical approval for the study was obtained from the ALSPAC Ethics and Law Committee and the Local Research Ethics Committees. Please note that the study website contains details of all the data that is available through a fully searchable data dictionary (<http://www.bris.ac.uk/alspac/researchers/data-access/data-dictionary/>).

The ALSPAC children and mothers were genotyped separately. ALSPAC children were genotyped on Illumina HumanHap550 quad-chip platforms by the Wellcome Trust Sanger Institute (Cambridge, UK) and by the Laboratory Corporation of America (Burlington, USA). ALSPAC mothers were genotyped on Illumina HumanHap660W quad-chip platform by Centre National de Génotypage (Évry, FR). Individuals were QC-filtered for: genotype missingness, X-chromosome mismatch, extreme autosomal heterozygosity, and cryptic relatedness (defined as IBD > 0.125). SNPs were QC-filtered for: excessive missingness, a significant ($p<1\times{10}^{-6}$) test of Hardy-Weinberg disequilibrium, and minor allele frequency under 1%.

The 465740 SNPs in common to mothers and children were used for phasing. Phasing was done with ShapeIt v2.r727 (Delaneau, Coulonges, and Zagury, 2008), based on a phased version of the 1000 genomes reference panel (Phase 1, Version 3), which we took from the Impute2 reference data repository (haplotype release date December 2013).

Using ancestry-informative principal components, we retained 5564 duos of European ancestry, which was reduced to 5558 duos giving consent to all measures. Across the 38 phenotypes which we analyzed, the numbers of duos with complete data differed slightly; the smallest subsample was 2136 duos (age at menarche), while the largest was 4753 duos (gestational age).

We used a *Perl* script to resolve the parent-of-origin of children's SNPs. The script requires mothers' and children's phased genotypes in *IMPUTE2* format (Marchini *et al.*, 2007). When parental origin is ambiguous (the child is heterozygous at a locus and the locus's membership in the two haplotypes are unclear), the alleles at the locus are assigned to the closest resolved haplotypes. The script outputs the phased genotypes with parent-of-origin assigned to *MACH*-formatted text files.

### Phenotyping Methods

#### PERINATAL MEASURES

Length of gestation was based on last menstrual period date, ultrasound assessment or other clinical indicators. Where there was conflict between the maternal report and ultrasound assessment, an experienced obstetrician reviewed the clinical records and made a best estimate. Birthweight was derived from obstetric data and from central birth notification data: where values disagreed by <100g then the lowest value was accepted; if the values disagreed by >100g then the value was coded as missing. Crown-heel length and head circumference of newborns were measured by trained staff a few days after birth. Ponderal index was calculated as birthweight (g) x 100 / (crown heel length (cm)^3).

#### ANTHROPOMETRIC MEASURES (7 year old clinic)

Height was measured to the nearest 0.1 cm using a Harpenden stadiometer (Holtain Ltd., Crymych, UK) and weight was measured to the nearest 50 g using Tanita weighing scales (Tanita UK Ltd, Uxbridge). Sitting Height was measured using the Harpenden sitting height table anthropometer to the last complete mm. The child was positioned on the table with back straight and thighs horizontal. Feet were supported on the footrest so that the knees were at right angles. Waist circumference was measured to the nearest mm at the minimum circumference of the abdomen between the iliac crests and the lowest ribs, the tape was kept perpendicular to the long axis of the body, touching the skin but not compressing the tissue. Hip circumference was measured to the nearest mm at the point of maximum circumference around the child’s hips/buttocks, again with the tape kept perpendicular to the long axis of the body segment. The measurement was done over the child’s pants. Body mass index was calculated as weight (in kg) divided by height (in m) squared. Waist hip ratio was calculated as waist circumference (in cm) divided by hip circumference (in cm).

#### BLOOD PRESSURE AND PULSE RATE (7 year old clinic)

Both blood pressure and pulse rates were measured using a Dinamap 9301 Vital Signs monitor. Two readings of systolic and diastolic blood pressure and pulse rates were recorded and averages taken.

#### HEMOGLOBIN (7 year old clinic)

Blood was sampled using an EDTA tube and hemoglobin levels were measured using the Haemocue system.

#### INTELLIGENCE (8 year old clinic)

The WISC-III UK was used to assess cognitive function. A short form of the measure was employed where alternate items (always starting with item number 1 in the standard form) were used for all subtests, with the exception of the coding subtest which was administered in its full form. Hence the length of the session was reduced and children were less likely to tire. All tests were administered by members of the psychology team. The ten WISC subtests comprised five verbal subtests:

1. Information (assessing the child’s knowledge);
2. Similarities (where similarities between things, e.g., in what way are red and blue alike? must be explained);
3. Arithmetic (comprising mental arithmetic questions);
4. Vocabulary (ascertaining the child’s understanding of the meaning of different words)
5. Comprehension (where the child is asked questions about different situations, e.g. why are names in the telephone book in alphabetical order?),

and five performance subtests:

1. Picture completion (where the child must point out what is missing from each of a series of pictures);
2. Coding (where shapes corresponding to different numbers must be copied as quickly as possible within a specified time limit);
3. Picture arrangement (where pictures must be ordered to make a meaningful sequence);
4. Block design (where pictures of specific patterns of blocks are copied with real blocks)
5. Object assembly (which involves putting together puzzles).

#### LUNG FUNCTION MEASURES (8 year old clinic)

American Thoracic Society (1995) criteria were used as the basis to select lung function measurements that were acceptable for analysis. Spirometers were calibrated at the beginning of each half-day session according to the manufacturer’s instructions using a 1L calibration syringe. Pneumotachograph screens were dried with warm air between subjects and cleaned at the end of each day’s testing, being allowed to dry overnight. Measurements were made in the sitting position without nose-clips. Children were instructed to fill their lungs completely and blow as hard and fast as possible until there was ‘no air’ left in their lungs. An on-screen incentive was used to encourage maximal expiratory effort (this comprised a visual of a fairground bell-and-hammer’ game – the object being to ring the bell). ‘Start of test’ criteria were automated within the Spirotrac programme and manoeuvres failing to meet these were rejected. Each subject was instructed to blow at least three times to produce a maximal expiratory manoeuvre. Repeatability criteria were set to three manoeuvres within 200ml FVC. Most children could not sustain forced expiration for the recommended 6s period. Curves were accepted if they reached a clear plateau of flow and the expiration had continued for >1 second and was judged by the tester to be a maximal effort. The best of three curves was selected for analysis (on the basis of an acceptable curve with the highest FVC measurement). All flow-volume curves were inspected post-hoc by a respiratory paediatrician (John Henderson) to ensure that satisfactory reproducibility criteria had been met and the optimal curve was selected for analysis.

Standard deviation scores adjusted for height, age and gender were calculated for FVC (the volume change of the lung between a full inspiration to total lung capacity and a maximal expiration to residual volume) and FEV1 (the volume exhaled during the first second of a forced expiratory manoeuvre started from the level of total lung capacity). The ratio of FEV1:FVC was calculated on the unadjusted values.

#### DXA DERIVED MEASURES (9 year old clinic)

Total body DXA scans were performed on all participants using a Lunar Prodigy scanner (Lunar Radiation Corp, Madison, WI) with paediatric scanning software (GE Healthcare Bio-Sciences Corp., Piscataway, NJ). DXA measures of BMD and bone area were derived for total body (less head). All DXA scans were subsequently reviewed by a trained researcher, and re-analysed as necessary, to ensure that borders between adjacent ROI’s were placed correctly by the automated software. The coefficient of variation for Total Body Less Head BMD measures was 0.8%, based on the analysis of 122 children who had two scans performed on the same day. Data on lean mass and fat mass (total body) were also obtained from the same scans.

#### BLOOD MEASURES (9 year old clinic)

Non-fasting blood samples were taken using standard procedures, with samples spun immediately and frozen at -80C. The measurements were assayed in 2008 after a median of 7.5 years in storage with no previous freeze-thaw cycles during this period Total plasma adiponectin concentrations were determined using ELISA (R&D Systems, Abingdon, UK), with the interassay coefficient of variation (CV) being 7%. Plasma lipids (i.e., total cholesterol, triglycerides, and high-density lipoprotein cholesterol) measured by a modification of the standard Lipid Research Clinics Protocol using enzymatic reagents for lipid determinations, leptin measured by an in-house ELISA validated against commercial methods, high-sensitivity interleukin 6 (IL-6) measured by ELISA (R&D Systems, Abingdon, UK), and C-reactive protein (CRP) measured by automated particle-enhanced immune-turbidimetric assay (Roche UK). All assay coefficients of variation were less than 5 % (Sayers et al. 2010).

#### 2D:4D RATIO (11 year old clinic)

Participants’ hands were photocopied during the eleven year old clinic visit, and measurements of the second and fourth fingers were taken from the photocopies with the use of digital calipers (accurate to 0.1 mm). The 2D:4D was calculated as the length of the second digit divided by the length of the fourth digit.

#### AGE AT MENARCHE

Age at menarche was derived from a series of nine postal questionnaires pertaining to pubertal development, sent approximately yearly from age 8 to 17 years. The questionnaires asked ‘Has your daughter started her menstrual periods yet?’ and, if yes: ‘How old was your daughter when she had her first period?’ Answers were given in years and months. These data were supplemented by questionnaires administered to girls at two research clinics attended at 12 years 10 months and 13 years 10 months asking ‘Have you started your periods yet?’ and, if yes: ‘When did you have your first period?’ The first-reported age at onset was used, as these results are least likely to be affected by recall bias (Sequeira et al. 2017).

#### TRANSFORMATIONS

The following variables were highly skewed and so subjected to inverse normal transformations before analysis: waist hip ratio, hip circumference, waist circumference, BMI, ponderal index, FEV1/FVC ratio, bone area, fat mass, lean mass, triglycerides, VLDL, LDL, HDL, IL6, CRP, Leptin and adiponectin.

# Supplementary Tables

## Table SI: Diagonal elements of ALSPAC GRMs

Summary statistics of the diagonal elements of GRMs of individuals in the ALSPAC data, for the additive ($A_{ii}$), dominance ($\Delta_{ii}$), and parent-of-origin effects ($\Gamma_{ii}$), respectively. The expected value for each is 1 in the absence of inbreeding.

| GRM | N | mean | sd | median | trimmed | mad | Min | max | range | skew | kurtosis |
| --- | --- | --- | --- | --- | --- | --- | --- | --- | --- | --- | --- |
| $A_{ii}$ | 4753 | 1.0 | 0.01 | 1 | 1.0 | 0.01 | 0.98 | 1.03 | 0.05 | 0.24 | 0.41 |
| $\Delta_{ii}$ | 4753 | 1.0 | 0.03 | 1 | 1.0 | 0.02 | 0.93 | 1.18 | 0.25 | 0.65 | 1.25 |
| $\Gamma_{ii}$ | 4753 | 0.98 | 0.01 | 0.98 | 0.98 | 0.01 | 0.96 | 1.02 | 0.06 | 0.19 | 0.45 |

## Table SII: Descriptive Statistics of 36 ALSPAC Phenotypes

| Phenotype | Units | N | Mean | SD | Min | Max | Mean Age(Months) | Age SD | Min Age | Mx Age |
| --- | --- | --- | --- | --- | --- | --- | --- | --- | --- | --- |
| 2D:4D  (Left hand) | - | 3847 | 0.966 | 0.032 | 0.844 | 1.087 | 140.781 | 2.721 | 127 | 163 |
| Adiponectin | ng/ml | 2972 | 13193.417 | 5490.027 | 90.368 | 43064.130 | - | - | - | - |
| Age at first tooth | months | 4262 | 6.989 | 2.364 | 1.000 | 16.000 | - | - | - | - |
| Age at Menarche | Years | 2136 | 12.673 | 1.145 | 8.417 | 16.667 | - | - | - | - |
| Birth Weight | G | 4689 | 3487.250 | 469.701 | 1752.000 | 5140.000 | - | - | - | - |
| Body Mass Index | - | 4065 | 16.187 | 2.001 | 10.848 | 29.686 | 89.997 | 3.133 | 82 | 113 |
| Bone Area | cm^2^ | 3858 | 1136.374 | 161.243 | 679.381 | 1820.254 | 118.213 | 3.602 | 105 | 138 |
| Bone Mineral Density | g/cm^2^ | 3858 | 0.777 | 0.053 | 0.603 | 0.982 | 118.213 | 3.602 | 105 | 138 |
| C-reactive Protein | mg/L | 2974 | 0.735 | 2.442 | 0.010 | 67.440 | - | - | - | - |
| Crown Heel Length | cm | 3848 | 50.910 | 2.145 | 42.000 | 59.000 | - | - | - | - |
| Diastolic Blood Pressure | mmHg | 4013 | 56.202 | 6.440 | 37.000 | 82.500 | 90.000 | 3.149 | 82 | 113 |
| Fat Mass | G | 3858 | 8489.803 | 4955.289 | 1268.194 | 34205.771 | 118.213 | 3.602 | 105 | 138 |
| FEV1 | Z score | 3624 | 0.021 | 0.979 | -3.768 | 3.782 | 103.467 | 3.331 | 89 | 124 |
| FEV1/FVC | - | 3625 | 0.885 | 0.066 | 0.501 | 1.000 | 103.467 | 3.330 | 89 | 124 |
| FVC | Z score | 3675 | 0.009 | 0.975 | -3.205 | 3.994 | 103.470 | 3.319 | 89 | 124 |
| Gestational Age | weeks | 4753 | 39.807 | 1.284 | 37.000 | 44.000 | - | - | - | - |
| HDL | mmol/L | 2974 | 1.400 | 0.307 | 0.440 | 2.680 | - | - | - | - |
| Head Circumference | cm | 3894 | 34.937 | 1.273 | 30.500 | 40.000 | - | - | - | - |
| Height | cm | 4069 | 125.861 | 5.546 | 104.400 | 148.200 | 89.992 | 3.116 | 82 | 113 |
| Hemoglobin | g/L | 3217 | 124.549 | 7.588 | 95.000 | 156.000 | 90.040 | 3.237 | 82 | 113 |
| Hip Circumference | cm | 4064 | 65.431 | 5.176 | 51.400 | 95.200 | 89.995 | 3.130 | 82 | 113 |
| Interleukin-6 | pg/ml | 2966 | 1.271 | 1.572 | 0.007 | 20.051 | - | - | - | - |
| LDL | mmol/L | 2974 | 2.353 | 0.595 | 0.563 | 9.184 | - | - | - | - |
| Lean Mass | g | 3858 | 24498.312 | 3172.135 | 15847.163 | 42012.328 | 118.213 | 3.602 | 105 | 138 |
| Leptin | ng/mL | 2973 | 8.253 | 8.109 | 0.900 | 92.000 | - | - | - | - |
| Number of teeth (15 months) | - | 4376 | 9.591 | 3.326 | 0.000 | 20.000 | - | - | - | - |
| Performance IQ | - | 3847 | 100.549 | 16.826 | 46.000 | 145.000 | 103.475 | 3.410 | 89 | 125 |
| Ponderal Index | gx100/cm^3^ | 3795 | 2.632 | 0.242 | 1.566 | 4.025 | - | - | - | - |
| Pulse Rate | Bpm | 4013 | 82.920 | 10.610 | 42.000 | 126.000 | 90.000 | 3.149 | 82 | 113 |
| Sitting Height | cm | 4068 | 67.990 | 2.773 | 59.200 | 78.800 | 89.998 | 3.132 | 82 | 113 |
| Systolic Blood Pressure | mmHg | 4016 | 98.640 | 9.067 | 66.500 | 135.000 | 89.998 | 3.147 | 82 | 113 |
| Total IQ | - | 3836 | 105.454 | 16.265 | 45.000 | 149.000 | 103.475 | 3.414 | 89 | 125 |
| Triglycerides | mmol/L | 2974 | 1.141 | 0.584 | 0.180 | 8.910 | - | - | - | - |
| Verbal IQ | - | 3850 | 108.536 | 16.638 | 50.000 | 155.000 | 103.477 | 3.423 | 89 | 125 |
| Waist-Hip Ratio | - | 4062 | 0.861 | 0.041 | 0.685 | 1.030 | 89.992 | 3.123 | 82 | 113 |
| Waist Circumference | cm | 4065 | 56.348 | 5.057 | 46.200 | 90.000 | 89.997 | 3.132 | 82 | 113 |

N: Number of

SD: Standard deviation

Min: Minimum

Max: Maximum

## Table SIII: GCTA Estimates of *G-REMLadp* models in ALSPAC

*Variance component estimates of 36 standardized phenotypes in ALSPAC, estimates and standard errors generated using GCTA-GREML to estimate multiple components simultaneously. VarA: proportion of phenotypic variance attributable to additive genetic effects; SEvarA: standard error of proportion of phenotypic variance attributable to additive genetic effects. VarD: proportion of phenotypic variance attributable to dominance effects; SEvarD: standard error of proportion of phenotypic variance attributable to dominance effects. VarP: proportion of phenotypic variance attributable to parent-of-origin effects; SEvarP: standard error of proportion of phenotypic variance attributable to parent-of-origin effects. Compare Table SIV for estimates fit using GCTA-GREML to fit single variance components and Table SVII for estimates based on unstandardized phenotypes.*

| Phenotype | VarA | SEvarA | VarD | SEvarD | VarP | SEvarP |
| --- | --- | --- | --- | --- | --- | --- |
| 2D:4D (Left hand) | 0.301 | 0.090 | -0.019 | 0.133 | -0.025 | 0.091 |
| Adiponectin | 0.140 | 0.117 | -0.123 | 0.169 | -0.039 | 0.118 |
| Age at first tooth | 0.425 | 0.083 | 0.114 | 0.121 | 0.156 | 0.082 |
| Age at Menarche | 0.337 | 0.162 | 0.195 | 0.246 | 0.179 | 0.162 |
| Birth Weight | 0.317 | 0.075 | -0.118 | 0.110 | 0.021 | 0.074 |
| Body Mass Index | 0.399 | 0.087 | -0.010 | 0.128 | 0.086 | 0.087 |
| Bone Area | 0.455 | 0.091 | -0.065 | 0.133 | 0.086 | 0.090 |
| Bone Mineral Density | 0.454 | 0.091 | -0.075 | 0.133 | 0.084 | 0.090 |
| C-reactive Protein | 0.165 | 0.115 | 0.119 | 0.172 | 0.068 | 0.119 |
| Crown Heel Length | 0.292 | 0.090 | -0.138 | 0.132 | 0.038 | 0.091 |
| Diastolic Blood Pressure | 0.193 | 0.088 | -0.076 | 0.132 | 0.114 | 0.088 |
| Fat Mass | 0.502 | 0.090 | 0.339 | 0.134 | 0.016 | 0.088 |
| FEV1 | 0.300 | 0.098 | -0.040 | 0.140 | 0.073 | 0.100 |
| FEV1/FVC | 0.376 | 0.097 | 0.060 | 0.143 | 0.018 | 0.096 |
| FVC | 0.370 | 0.097 | 0.017 | 0.136 | 0.159 | 0.097 |
| Gestational Age | 0.175 | 0.074 | 0.132 | 0.112 | -0.055 | 0.072 |
| HDL | 0.114 | 0.114 | -0.201 | 0.171 | -0.024 | 0.117 |
| Head Circumference | 0.433 | 0.089 | 0.042 | 0.134 | -0.095 | 0.088 |
| Height | 0.468 | 0.088 | 0.029 | 0.128 | -0.146 | 0.082 |
| Hemoglobin | 0.423 | 0.107 | 0.274 | 0.159 | 0.038 | 0.108 |
| Hip Circumference | 0.507 | 0.088 | -0.026 | 0.129 | 0.060 | 0.086 |
| Interleukin-6 | 0.115 | 0.119 | 0.255 | 0.174 | 0.092 | 0.118 |
| LDL | 0.124 | 0.117 | -0.184 | 0.173 | 0.072 | 0.118 |
| Lean Mass | 0.356 | 0.091 | 0.133 | 0.138 | 0.009 | 0.091 |
| Leptin | 0.416 | 0.117 | 0.018 | 0.170 | 0.041 | 0.115 |
| Number of teeth (15 months) | 0.325 | 0.082 | -0.011 | 0.117 | 0.019 | 0.079 |
| Performance IQ | 0.209 | 0.090 | 0.230 | 0.135 | -0.029 | 0.091 |
| Ponderal Index | 0.237 | 0.094 | 0.005 | 0.137 | -0.018 | 0.090 |
| Pulse Rate | 0.112 | 0.087 | -0.044 | 0.128 | -0.051 | 0.084 |
| Sitting Height | 0.450 | 0.087 | 0.085 | 0.127 | -0.111 | 0.083 |
| Systolic Blood Pressure | 0.313 | 0.088 | -0.147 | 0.130 | 0.145 | 0.088 |
| Total IQ | 0.357 | 0.089 | 0.260 | 0.134 | -0.142 | 0.088 |
| Triglycerides | 0.013 | 0.115 | -0.063 | 0.171 | -0.057 | 0.120 |
| Verbal IQ | 0.440 | 0.089 | 0.295 | 0.133 | -0.098 | 0.089 |
| Waist-Hip Ratio | 0.100 | 0.085 | -0.242 | 0.124 | -0.022 | 0.085 |
| Waist Circumference | 0.450 | 0.088 | -0.091 | 0.127 | -0.019 | 0.085 |

## Table SIV: Single-component GCTA Estimates of *G-REMLadp* models in ALSPAC

*Standardized variance component estimates of 36 phenotypes in ALSPAC generated using three runs of GCTA-GREML, fitting single components each time. VarA: proportion of phenotypic variance attributable to additive genetic effects; SEvarA: standard error of proportion of phenotypic variance attributable to additive genetic effects. VarD: proportion of phenotypic variance attributable to dominance effects; SEvarD: standard error of proportion of phenotypic variance attributable to dominance effects. VarP: proportion of phenotypic variance attributable to parent-of-origin effects; SEvarP: standard error of proportion of phenotypic variance attributable to parent-of-origin effects. Compare Table SIII for estimates fit using GCTA-GREML to fit multiple variance components.*

| Phenotype | VarA | SEvarA | VarD | SEvarD | VarP | SEvarP |
| --- | --- | --- | --- | --- | --- | --- |
| 2D:4D (Left hand) | 0.302 | 0.090 | -0.036 | 0.134 | -0.032 | 0.092 |
| Adiponectin | 0.142 | 0.117 | -0.128 | 0.169 | -0.040 | 0.118 |
| Age at first tooth | 0.415 | 0.084 | 0.097 | 0.123 | 0.138 | 0.083 |
| Age at Menarche | 0.334 | 0.162 | 0.198 | 0.248 | 0.170 | 0.163 |
| Birth Weight | 0.312 | 0.075 | -0.092 | 0.111 | 0.008 | 0.074 |
| Body Mass Index | 0.396 | 0.088 | -0.029 | 0.130 | 0.068 | 0.088 |
| Bone Area | 0.449 | 0.092 | -0.036 | 0.136 | 0.059 | 0.091 |
| Bone Mineral Density | 0.448 | 0.092 | -0.048 | 0.135 | 0.057 | 0.091 |
| C-reactive Protein | 0.169 | 0.115 | 0.120 | 0.172 | 0.077 | 0.119 |
| Crown Heel Length | 0.293 | 0.090 | -0.141 | 0.133 | 0.030 | 0.092 |
| Diastolic Blood Pressure | 0.191 | 0.088 | -0.045 | 0.132 | 0.116 | 0.088 |
| Fat Mass | 0.502 | 0.090 | 0.339 | 0.136 | 0.025 | 0.090 |
| FEV1 | 0.299 | 0.099 | -0.040 | 0.141 | 0.070 | 0.100 |
| FEV1/FVC | 0.376 | 0.098 | 0.052 | 0.144 | 0.028 | 0.097 |
| FVC | 0.371 | 0.097 | 0.038 | 0.138 | 0.164 | 0.098 |
| Gestational Age | 0.174 | 0.074 | 0.127 | 0.113 | -0.056 | 0.072 |
| HDL | 0.112 | 0.114 | -0.199 | 0.171 | -0.038 | 0.117 |
| Head Circumference | 0.436 | 0.089 | 0.036 | 0.136 | -0.109 | 0.088 |
| Height | 0.474 | 0.088 | 0.036 | 0.129 | -0.163 | 0.083 |
| Hemoglobin | 0.437 | 0.107 | 0.330 | 0.161 | 0.055 | 0.109 |
| Hip Circumference | 0.505 | 0.088 | -0.024 | 0.131 | 0.041 | 0.087 |
| Interleukin-6 | 0.116 | 0.120 | 0.264 | 0.174 | 0.094 | 0.118 |
| LDL | 0.119 | 0.117 | -0.176 | 0.173 | 0.080 | 0.119 |
| Lean Mass | 0.359 | 0.091 | 0.152 | 0.139 | -0.019 | 0.091 |
| Leptin | 0.417 | 0.117 | 0.061 | 0.171 | 0.039 | 0.116 |
| Number of teeth (15 months) | 0.326 | 0.082 | -0.028 | 0.117 | 0.021 | 0.079 |
| Performance IQ | 0.218 | 0.090 | 0.246 | 0.135 | -0.031 | 0.092 |
| Ponderal Index | 0.238 | 0.094 | 0.029 | 0.138 | -0.025 | 0.090 |
| Pulse Rate | 0.110 | 0.087 | -0.044 | 0.128 | -0.051 | 0.084 |
| Sitting Height | 0.453 | 0.087 | 0.066 | 0.128 | -0.129 | 0.083 |
| Systolic Blood Pressure | 0.303 | 0.088 | -0.085 | 0.131 | 0.150 | 0.089 |
| Total IQ | 0.364 | 0.089 | 0.281 | 0.135 | -0.158 | 0.089 |
| Triglycerides | 0.013 | 0.115 | -0.066 | 0.171 | -0.059 | 0.120 |
| Verbal IQ | 0.441 | 0.089 | 0.296 | 0.134 | -0.122 | 0.089 |
| Waist-Hip Ratio | 0.097 | 0.086 | -0.239 | 0.124 | -0.021 | 0.086 |
| Waist Circumference | 0.450 | 0.088 | -0.080 | 0.130 | -0.029 | 0.086 |

## Table SV: HE Regression Estimates of G-REMLadp models in ALSPAC

*Variance component estimates of 36 standardized phenotypes in ALSPAC, estimates and standard errors generated with single-components HE regression. VarA: proportion of phenotypic variance attributable to additive genetic effects; SEvarA: standard error of proportion of phenotypic variance attributable to additive genetic effects. VarD: proportion of phenotypic variance attributable to dominance effects; SEvarD: standard error of proportion of phenotypic variance attributable to dominance effects. VarP: proportion of phenotypic variance attributable to parent-of-origin effects; SEvarP: standard error of proportion of phenotypic variance attributable to parent-of-origin effects. Compare Table SIII for estimates fit using GCTA-GREML to estimate variance components.*

| Phenotype | VarA | SEvarA | VarD | SEvarD | VarP | SEvarP |
| --- | --- | --- | --- | --- | --- | --- |
| 2D:4D (Left hand) | 0.298 | 0.136 | -0.010 | 0.191 | -0.039 | 0.124 |
| Adiponectin | 0.135 | 0.165 | -0.089 | 0.251 | -0.067 | 0.167 |
| Age at first tooth | 0.402 | 0.115 | 0.099 | 0.175 | 0.134 | 0.118 |
| Age at Menarche | 0.321 | 0.228 | 0.112 | 0.332 | 0.169 | 0.236 |
| Birth Weight | 0.294 | 0.104 | -0.104 | 0.152 | -0.008 | 0.108 |
| Body Mass Index | 0.383 | 0.121 | -0.018 | 0.180 | 0.070 | 0.121 |
| Bone Area | 0.475 | 0.129 | -0.006 | 0.186 | 0.049 | 0.126 |
| Bone Mineral Density | 0.475 | 0.129 | -0.016 | 0.186 | 0.046 | 0.126 |
| C-reactive Protein | 0.132 | 0.160 | 0.206 | 0.248 | 0.073 | 0.165 |
| Crown Heel Length | 0.291 | 0.133 | -0.106 | 0.187 | -0.004 | 0.127 |
| Diastolic Blood Pressure | 0.178 | 0.118 | -0.012 | 0.183 | 0.117 | 0.125 |
| **Fat Mass** | **0.483** | **0.089** | **0.384** | **0.135** | **0.013** | **0.091** |
| FEV1 | 0.274 | 0.138 | -0.064 | 0.201 | 0.058 | 0.136 |
| FEV1/FVC | 0.372 | 0.135 | 0.032 | 0.198 | 0.010 | 0.138 |
| FVC | 0.341 | 0.134 | 0.020 | 0.207 | 0.154 | 0.135 |
| Gestational Age | 0.180 | 0.102 | 0.130 | 0.149 | -0.063 | 0.107 |
| HDL | 0.118 | 0.171 | -0.189 | 0.244 | -0.050 | 0.164 |
| Head Circumference | 0.430 | 0.134 | -0.181 | 0.133 | -0.097 | 0.128 |
| Height | 0.448 | 0.121 | -0.015 | 0.177 | -0.176 | 0.123 |
| Hemoglobin | 0.473 | 0.156 | 0.317 | 0.237 | 0.066 | 0.160 |
| Hip Circumference | 0.425 | 0.120 | -0.030 | 0.174 | 0.048 | 0.124 |
| Interleukin-6 | 0.071 | 0.161 | 0.258 | 0.250 | 0.091 | 0.170 |
| LDL | 0.118 | 0.157 | -0.139 | 0.246 | 0.025 | 0.163 |
| Lean Mass | 0.388 | 0.128 | 0.135 | 0.189 | -0.014 | 0.129 |
| Leptin | 0.362 | 0.168 | 0.151 | 0.256 | 0.013 | 0.168 |
| Number of teeth (15 months) | 0.301 | 0.110 | -0.043 | 0.171 | 0.011 | 0.113 |
| Performance IQ | 0.221 | 0.130 | 0.244 | 0.192 | -0.032 | 0.124 |
| Ponderal Index | 0.216 | 0.126 | 0.013 | 0.197 | -0.027 | 0.135 |
| Pulse Rate | 0.105 | 0.123 | -0.032 | 0.183 | -0.057 | 0.126 |
| Sitting Height | 0.435 | 0.121 | 0.052 | 0.180 | -0.146 | 0.125 |
| Systolic Blood Pressure | 0.300 | 0.124 | -0.051 | 0.179 | 0.147 | 0.121 |
| Total IQ | 0.400 | 0.136 | 0.261 | 0.192 | -0.162 | 0.128 |
| Triglycerides | 0.022 | 0.167 | -0.059 | 0.248 | -0.047 | 0.165 |
| Verbal IQ | 0.484 | 0.138 | 0.286 | 0.196 | -0.128 | 0.129 |
| Waist-Hip Ratio | 0.021 | 0.118 | -0.233 | 0.174 | -0.005 | 0.122 |
| Waist Circumference | 0.425 | 0.120 | -0.070 | 0.180 | -0.037 | 0.124 |

## Table SVI: Accuracy of $\boldsymbol{\chi}^{\boldsymbol{2}}$ approximations across all simulations

Accuracy of $\chi^{2}$ approximations to noncentral $F$, measured as percent relative difference ("%Diff") between the average test statistic observed in the simulations and the expected value. This has been computed over all simulation replicates performed for this study. N: Number of simulated phased genotypes; Parent Corr: average correlation of simulated parental genotypes; MAF Diff: average difference in simulated parental MAFs; #Reps: number of simulated replications; POE Wald %Diff: percent relative difference between the observed 1-df Wald test statistic for HE regression of parent-of-origin variance components and the expected value, averaged across simulated replications; HE $F$ %Diff is 3-df $F$ test is the percent relative difference between the 3-df Wald test of additive, dominance, and parent-of-origin variance components in Haseman-Elston regression and the expected value, averaged across simulated replications; REML LRT %Diff: the percent relative difference between the 3-df $\chi^{2}$ test of additive, dominance, and parent-of-origin variance components from REML fitting using GCTA and the expected value, averaged across simulated replications.

| N | Parent Corr | MAF Diff | #Reps | POE Wald %Diff | HE $F$ Diff | REML LRT Diff |
| --- | --- | --- | --- | --- | --- | --- |
| 1000 | 0.00 | 0.00 | 2727 | 4.629 | -11.660 | -10.550 |
| 1000 | 0.00 | 0.05 | 900 | 1.371 | -40.240 | -41.590 |
| 1000 | 0.25 | 0.00 | 876 | -17.770 | 58.080 | -32.670 |
| 1000 | 0.25 | 0.05 | 504 | -16.660 | -23.200 | -37.190 |
| 2000 | 0.00 | 0.00 | 2775 | -0.058 | -19.010 | -16.100 |
| 2000 | 0.00 | 0.05 | 900 | 1.292 | -55.990 | -60.040 |
| 2000 | 0.25 | 0.00 | 900 | -32.830 | 60.010 | -48.060 |
| 2000 | 0.25 | 0.05 | 876 | -30.460 | -29.630 | -48.330 |
| 4000 | 0.00 | 0.00 | 2675 | 2.240 | -21.770 | -22.980 |
| 4000 | 0.00 | 0.05 | 888 | -2.641 | -63.860 | -66.920 |
| 4000 | 0.25 | 0.00 | 600 | -45.610 | 51.890 | -57.480 |
| 4000 | 0.25 | 0.05 | 600 | -43.710 | -35.000 | -56.030 |
| 5000 | 0.00 | 0.00 | 280 | 2.702 | 1.745 | 1.752 |
| 5000 | 0.00 | 0.05 | 280 | -2.181 | -1.452 | -5.536 |
| 7500 | 0.00 | 0.00 | 250 | -10.930 | -4.248 | 0.628 |
| 7500 | 0.00 | 0.05 | 240 | 6.520 | 6.118 | 12.700 |

## Table SVII: GCTA Estimates of *G-REMLadp* models of unstandardized phenotypes in ALSPAC

*Variance component estimates of 36 unstandardized phenotypes in ALSPAC, estimates and standard errors generated using GCTA-GREML to estimate multiple components simultaneously. VarA: proportion of phenotypic variance attributable to additive genetic effects; SEvarA: standard error of proportion of phenotypic variance attributable to additive genetic effects. VarD: proportion of phenotypic variance attributable to dominance effects; SEvarD: standard error of proportion of phenotypic variance attributable to dominance effects. VarP: proportion of phenotypic variance attributable to parent-of-origin effects; SEvarP: standard error of proportion of phenotypic variance attributable to parent-of-origin effects. VarE: estimate of phenotypic error variance; SEvarE: standard error of estimate of phenotypic error variance. PhVar: Estimate of phenotypic variance. PhSE: Standard error of phenotypic variance estimate. Compare Table SIV for estimates based on standardized phenotypes. See Table SIII for descriptive statistics for each phenotype.*

| Phenotype | VarA | SEvarA | VarD | SEvarD | VarP | SEvarP | VarE | SEvarE | PhVar | PhSE |
| --- | --- | --- | --- | --- | --- | --- | --- | --- | --- | --- |
| 2D:4D  (Left hand) | 3.0x10^-4^ | 9.2x10^-5^ | -2.0x10^-5^ | 1.4x10^-5^ | -2.5x10^-5^ | 9.2x10^-5^ | 7.5x10^-4^ | 1.9x10^-4^ | 1.0x10^-3^ | 2.3x10^-5^ |
| Adiponectin | 0.136 | 0.114 | -0.120 | 0.166 | -0.038 | 0.115 | 0.998 | 0.234 | 0.976 | 0.025 |
| Age at first tooth | 2.376 | 0.474 | 0.636 | 0.678 | 0.873 | 0.459 | 1.700 | 0.938 | 5.585 | 0.122 |
| Age at Menarche | 0.443 | 0.214 | 0.257 | 0.324 | 0.236 | 0.214 | 0.380 | 0.440 | 1.316 | 0.041 |
| Birth Weight | 68668.472 | 16478.140 | -25620.92 | 23774.703 | 4548.509 | 15960.585 | 169126.413 | 32808.301 | 216722.430 | 4506.383 |
| Body Mass Index | 0.382 | 0.085 | -0.009 | 0.123 | 0.082 | 0.083 | 0.504 | 0.172 | 0.959 | 0.021 |
| Bone Area | 0.430 | 0.088 | -0.062 | 0.126 | 0.082 | 0.085 | 0.495 | 0.175 | 0.945 | 0.022 |
| Bone Mineral Density | 1.3x10^-3^ | 2.6x10^-4^ | -2.1x10^-4^ | 3.7x10^-4^ | 2.4x10^-4^ | 2.5x10^-4^ | 1.5x10^-3^ | 5.2x10^-4^ | 2.8x10^-3^ | 6.5x10^-5^ |
| C-reactive Protein | 0.153 | 0.107 | 0.111 | 0.160 | 0.064 | 0.111 | 0.603 | 0.219 | 0.931 | 0.024 |
| Crown Heel Length | 1.298 | 0.404 | -0.612 | 0.586 | 0.168 | 0.407 | 3.588 | 0.819 | 4.443 | 0.102 |
| Diastolic BP | 7.978 | 3.654 | -3.131 | 5.461 | 4.704 | 3.649 | 31.842 | 7.346 | 41.394 | 0.930 |
| Fat Mass | 0.441 | 0.081 | 0.297 | 0.118 | 0.014 | 0.077 | 0.126 | 0.159 | 0.878 | 0.020 |
| FEV1 | 0.288 | 0.095 | -0.039 | 0.135 | 0.070 | 0.096 | 0.642 | 0.190 | 0.961 | 0.023 |
| FEV1/FVC | 0.371 | 0.098 | 0.059 | 0.141 | 0.018 | 0.095 | 0.540 | 0.195 | 0.988 | 0.023 |
| FVC | 0.353 | 0.094 | 0.016 | 0.130 | 0.152 | 0.093 | 0.434 | 0.180 | 0.954 | 0.022 |
| Gest. Age | 0.289 | 0.122 | 0.217 | 0.185 | -0.090 | 0.118 | 1.229 | 0.251 | 1.644 | 0.034 |
| HDL | 0.110 | 0.110 | -0.194 | 0.166 | -0.024 | 0.113 | 1.073 | 0.226 | 0.965 | 0.025 |
| Head Circumference | 0.650 | 0.136 | 0.063 | 0.201 | -0.143 | 0.131 | 0.931 | 0.275 | 1.501 | 0.034 |
| Height | 14.298 | 2.736 | 0.899 | 3.895 | -4.448 | 2.507 | 19.772 | 5.357 | 30.520 | 0.682 |
| Hemoglobin | 24.284 | 6.254 | 15.722 | 9.131 | 2.203 | 6.181 | 15.242 | 12.208 | 57.451 | 1.444 |
| Hip Circumference | 0.472 | 0.084 | -0.024 | 0.120 | 0.056 | 0.080 | 0.427 | 0.166 | 0.931 | 0.021 |
| Interleukin-6 | 0.115 | 0.119 | 0.253 | 0.173 | 0.091 | 0.117 | 0.533 | 0.237 | 0.992 | 0.026 |
| LDL | 0.119 | 0.113 | -0.177 | 0.166 | 0.069 | 0.114 | 0.949 | 0.228 | 0.960 | 0.025 |
| Lean Mass | 0.310 | 0.080 | 0.116 | 0.121 | 0.008 | 0.079 | 0.437 | 0.165 | 0.870 | 0.020 |
| Leptin | 0.359 | 0.102 | 0.015 | 0.147 | 0.035 | 0.099 | 0.453 | 0.200 | 0.862 | 0.023 |
| Number of teeth (15 months) | 3.597 | 0.919 | -0.120 | 1.292 | 0.211 | 0.869 | 7.368 | 1.808 | 11.056 | 0.238 |
| Perform. IQ | 59.208 | 25.498 | 64.957 | 38.142 | -8.068 | 25.794 | 166.771 | 51.841 | 282.867 | 6.474 |
| Ponderal Index | 1.4x10^-2^ | 5.5x10^-3^ | 3.0x10^-4^ | 8.0x10^-3^ | -1.1x10^-3^ | 5.2x10^-3^ | 4.5x10^-2^ | 1.1x10^-2^ | 0.059 | 1.4x10^-3^ |
| Pulse Rate | 12.366 | 9.662 | -4.904 | 14.185 | -5.660 | 9.340 | 108.920 | 19.315 | 110.723 | 2.477 |
| Sitting Height | 3.408 | 0.675 | 0.641 | 0.960 | -0.844 | 0.627 | 4.373 | 1.331 | 7.578 | 0.169 |
| Systolic BP | 25.804 | 7.294 | -12.141 | 10.707 | 11.940 | 7.307 | 56.874 | 14.479 | 82.477 | 1.858 |
| Total IQ | 94.213 | 23.878 | 68.501 | 35.524 | -37.494 | 23.310 | 138.613 | 48.441 | 263.833 | 6.056 |
| Triglycerides | 0.013 | 0.114 | -0.062 | 0.169 | -0.056 | 0.119 | 1.098 | 0.236 | 0.993 | 0.026 |
| Verbal IQ | 121.119 | 25.017 | 81.285 | 36.742 | -26.993 | 24.417 | 100.077 | 50.481 | 275.488 | 6.328 |
| Waist-Hip Ratio | 1.5x10^-4^ | 1.3x10^-4^ | -3.5x10^-4^ | 1.8x10^-4^ | -3.2x10^-5^ | 1.2x10^-4^ | 1.7x10^-3^ | 2.6x10^-4^ | 1.5x10^-3^ | 3.2x10^-5^ |
| Waist Circumference | 0.423 | 0.084 | -0.086 | 0.120 | -0.018 | 0.080 | 0.620 | 0.166 | 0.939 | 0.021 |

# Supplementary Figures

## S1: *Scatterplot matrix of off diagonal elements of ALSPAC GRMs*


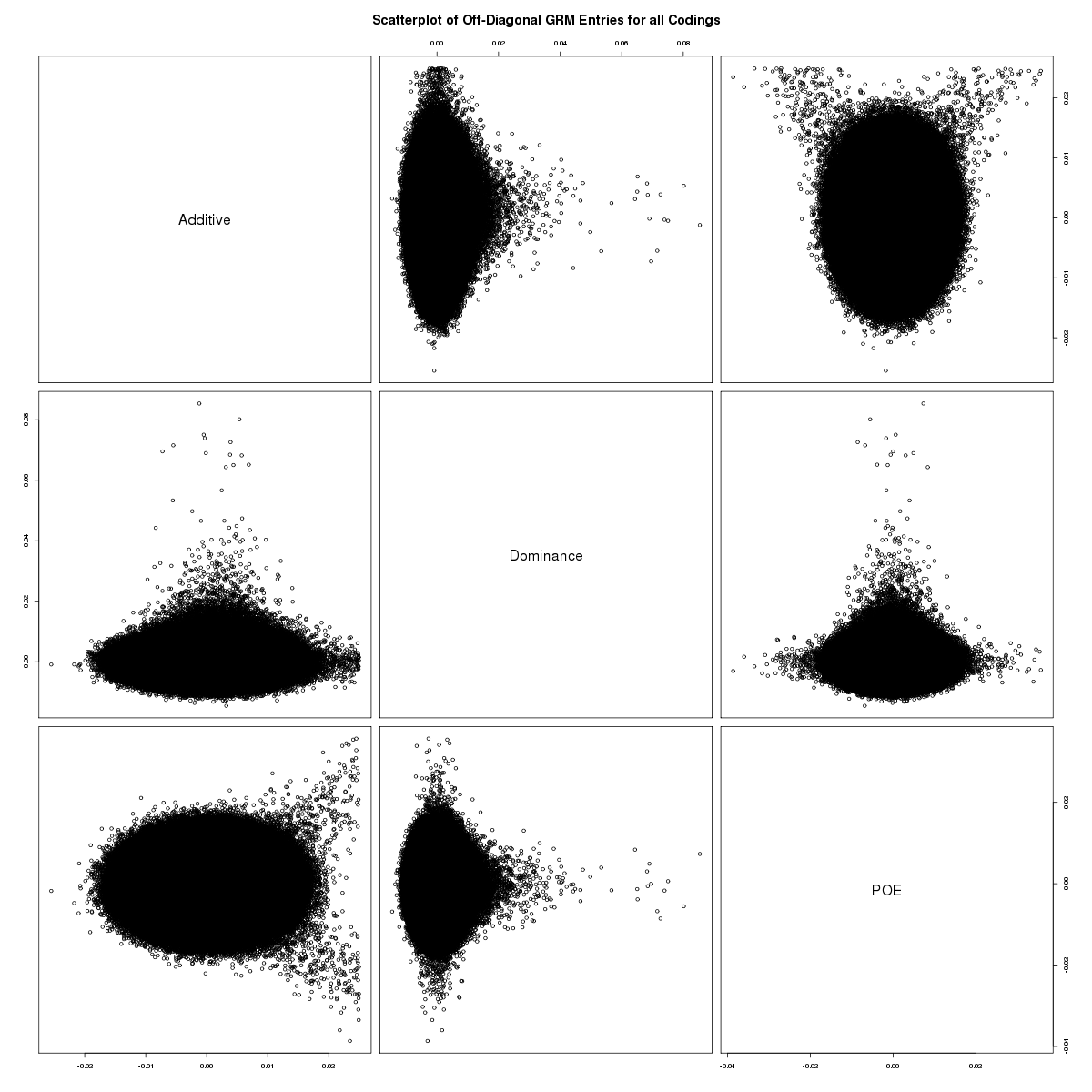
*Scatterplot matrix of off diagonal elements of genetic relationship matrices for additive, dominance, and parent-of-origin effects*, *taken from the analysis of gestational age in ALSPAC. See Tables SII, SIII, and SVII for descriptive statistics and G-REMLadp model results from this analysis.*

## S2: Expected power to detect parent-of-origin effects tagged by $\boldsymbol{m=10000}$ loci

**Expected power to detect parent-of-origin effects tagged by** $m=10000$ **loci, by sample size**: Curves generated using Haseman-Elston regression approximation to the Wald test of nonzero effect. %PoE: proportion of phenotypic variance attributable to parent-of-origin effects; Sample size: number of unrelated individuals with phased genotypes in the sample that would be used to estimate POEs; Power: proportion of test statistics generated under a non-null distribution with noncentrality parameter as given in Equation 4 that would exceed a 95% critical value in the Wald test of a null variance component.


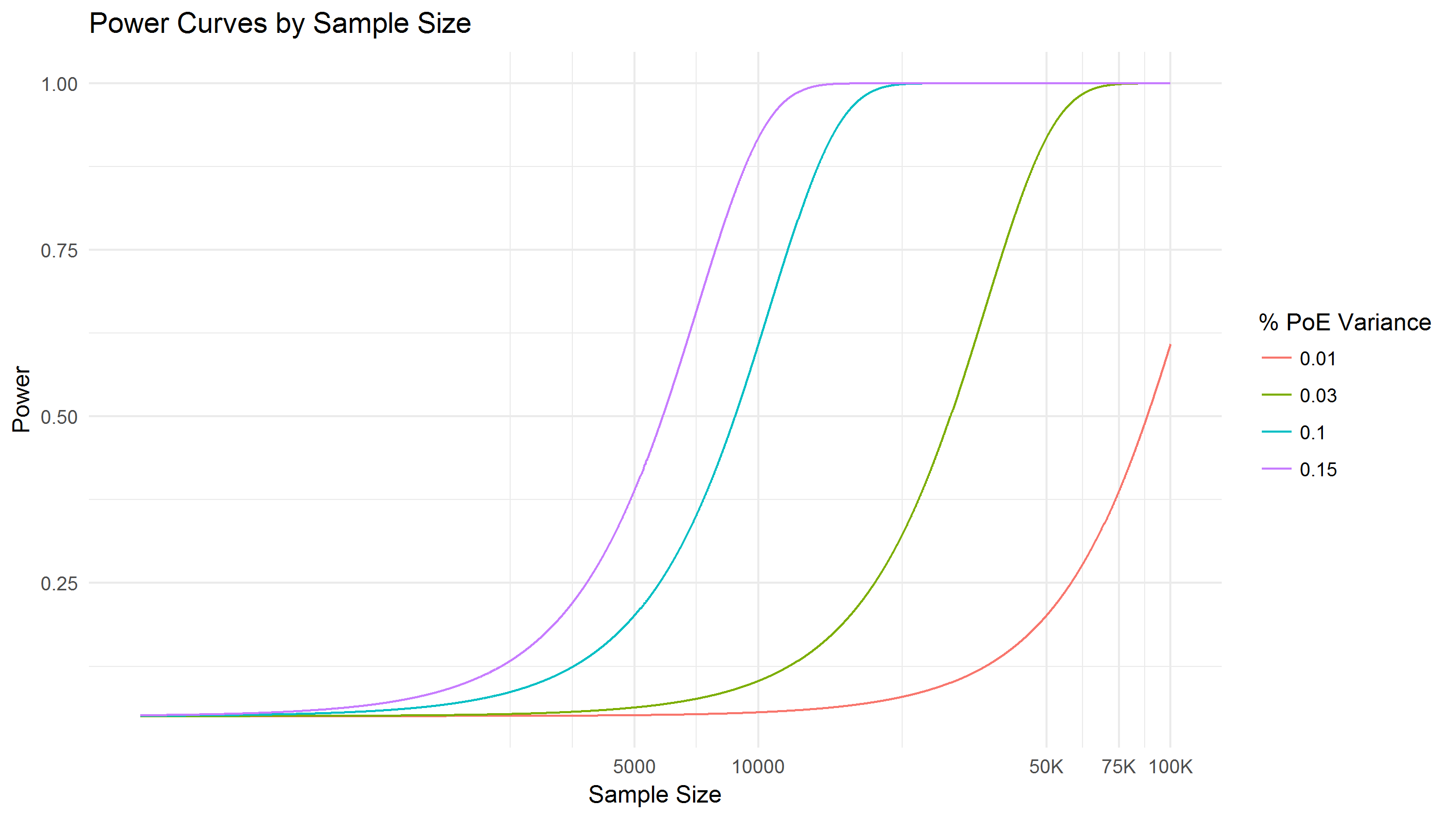


## S3: *Wald test statistics for simulated additive effects*

*Observed Wald test statistics for simulated additive effects, averaged over replications, versus their expected values*. Averaged observed $\chi^{2}$ is the mean Wald test statistic over replications, within a particular simulated condition; Expected $\chi^{2}$ is the expected Wald test statistic for that effect size; Correlation between parental genotypes: the average correlation between maternal and paternal transmitted genotypes, circles being no correlation, triangles low correlation, $r=0.025$, and squares high correlation, $r=0.25$; Sample size, coded by point colour is the number of simulated children with phased, parent-of-origin determined genotypes; Variance component size, coded by point size, is the population value of the proportion of phenotypic variance explained by the simulated additive effects. The black line is the line of equality.


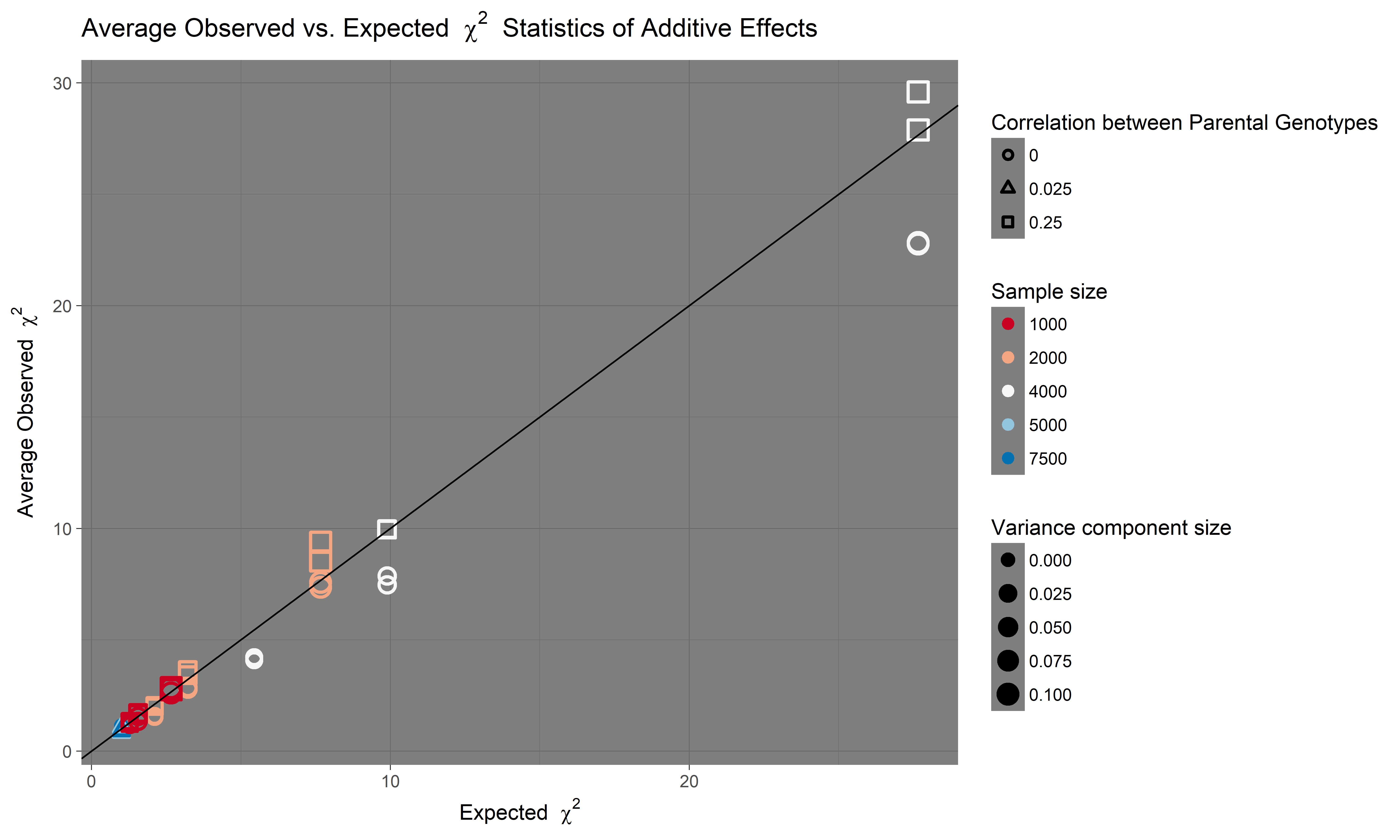


## S4: *Wald test statistics for simulated dominance effects*

*Observed Wald test statistics for simulated dominance effects, averaged over replications, versus their expected values*. Averaged observed $\chi^{2}$ is the mean Wald test statistic over replications, within a particular simulated condition; Expected $\chi^{2}$ is the expected Wald test statistic for that effect size; Correlation between parental genotypes: the average correlation between maternal and paternal transmitted genotypes, circles being no correlation, triangles low correlation, $r=0.025$, and squares high correlation, $r=0.25$; Sample size, coded by point colour is the number of simulated children with phased, parent-of-origin determined genotypes; Variance component size, coded by point size, is the population value of the proportion of phenotypic variance explained by the simulated additive effects. The black line is the line of equality.


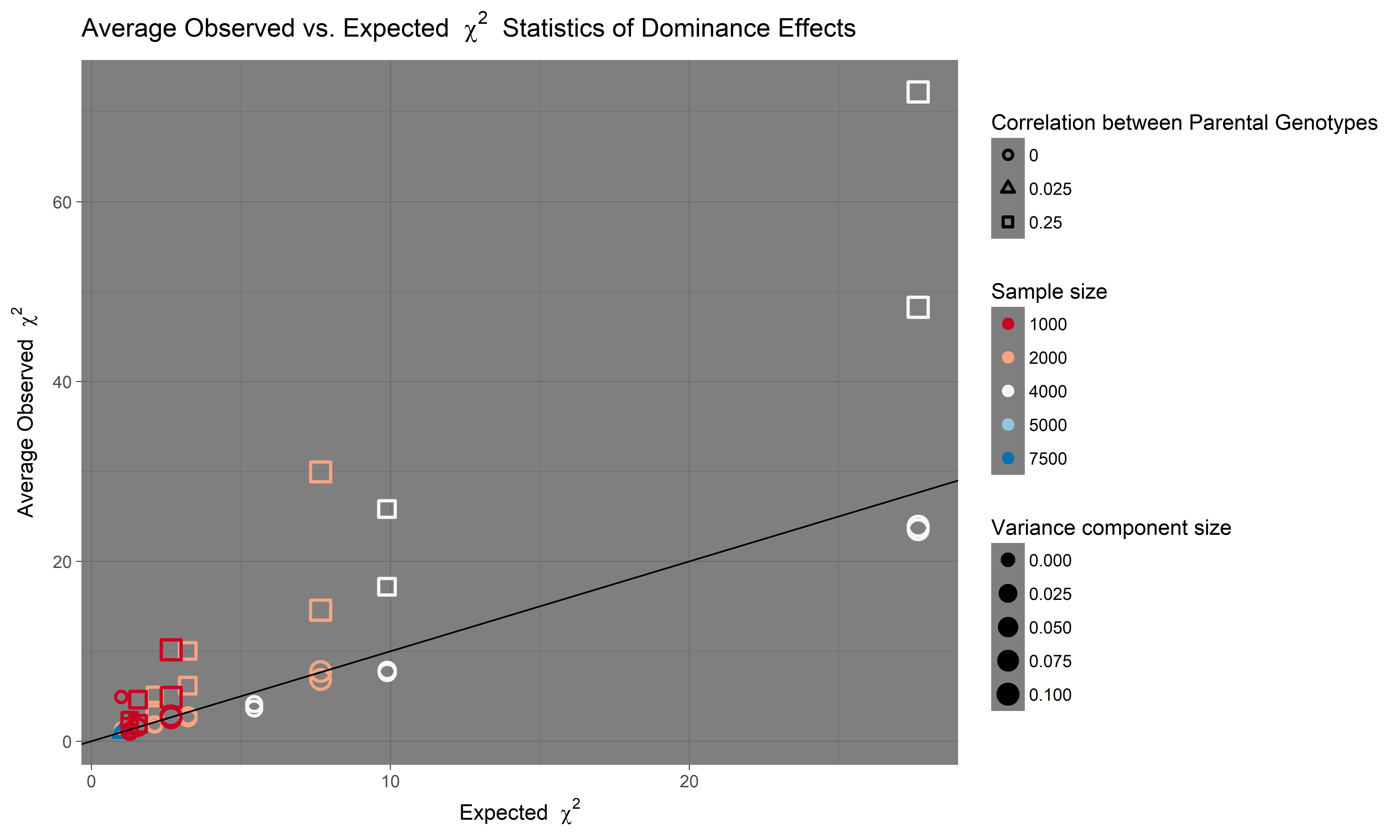


## S5: *Wald test statistics for simulated parent-of-origin effects*

*Observed Wald test statistics for simulated parent-of-origin effects (POE), averaged over replications, versus their expected values*. Averaged observed $\chi^{2}$ is the mean Wald test statistic over replications, within a particular simulated condition; Expected $\chi^{2}$ is the expected Wald test statistic for that effect size; Correlation between parental genotypes: the average correlation between maternal and paternal transmitted genotypes, circles being no correlation, triangles low correlation, $r=0.025$, and squares high correlation, $r=0.25$; Sample size, coded by point colour is the number of simulated children with phased, parent-of-origin determined genotypes; Variance component size, coded by point size, is the population value of the proportion of phenotypic variance explained by the simulated additive effects. The black line is the line of equality.


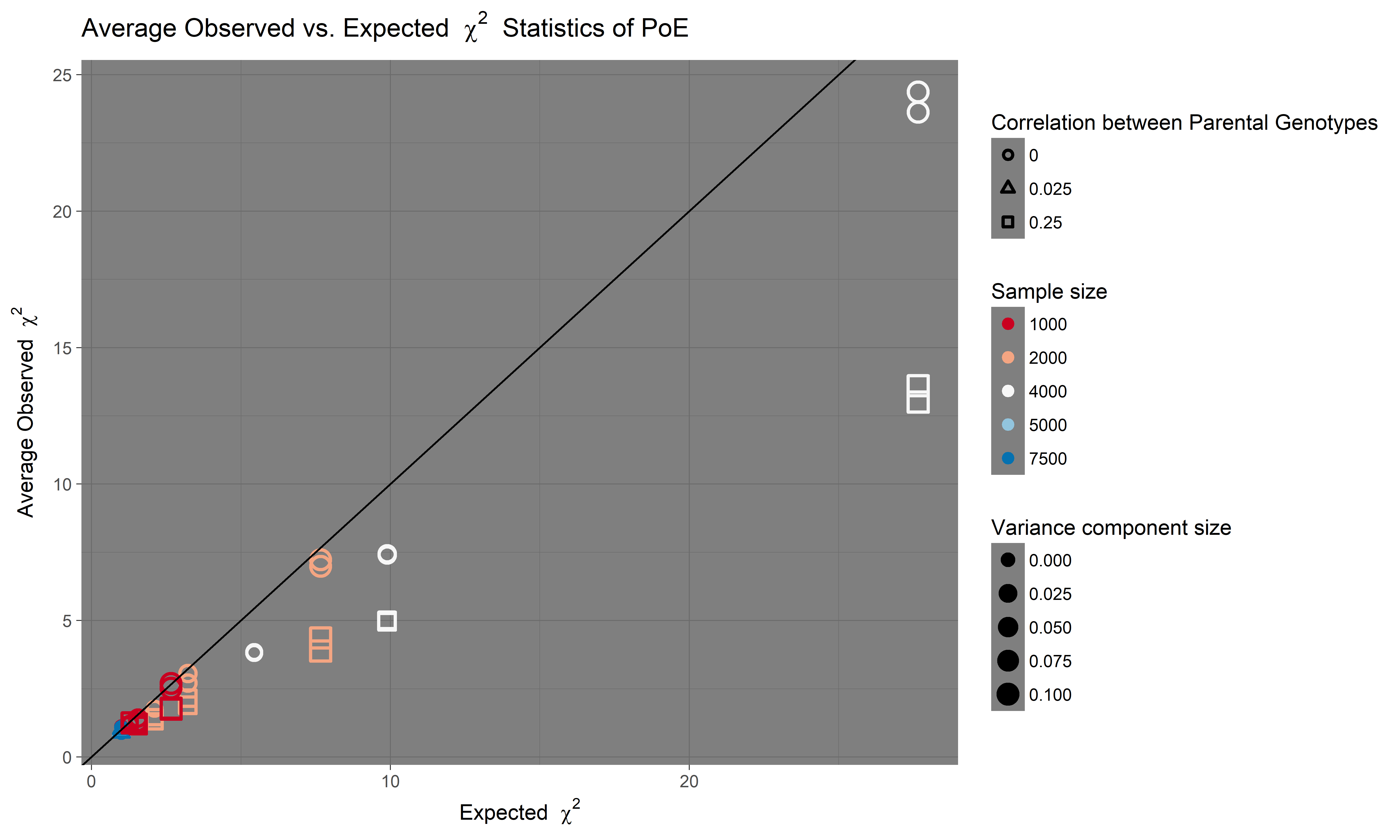


# Appendix: Correlations between codings of phased genotypes at two loci

## Notation and Parental gametes, considering two linked loci

Consider loci I and II, which are in linkage disequilibrium. Represent the four transmitted alleles at these loci by four random variables: for the first locus, $\tau_{I,Mo}, \tau_{I,Fa}$ and $\tau_{II,Mo}, \tau_{II,Fa}$ at the second locus, where

$$\tau=\left\{ \begin{matrix} 1 & if minor allele transmitted \\ 0 & if major allele transmitted \end{matrix} \right.$$

We assume: random mating so that $\tau_{.,Mo}$ are independent of $\tau_{.,Fa}$, and equal allele frequencies across sexes. For simplicity, assume that the minor allele frequencies(MAFs) are equal across loci; the results here can be extended straightforwardly to loci with differing MAFs.

With MAF equal to $p$ and defining $q=1-p$:

- $E\left( \tau_{I,Mo} \right)=E\left( \tau_{I,Fa} \right)=E\left( \tau_{II,Mo} \right)=E\left( \tau_{II,Fa} \right)=p$
- Because the $\tau$ are indicator variables, $E\left( \tau_{I,Mo}^{2} \right)=E\left( \tau_{I,Fa}^{2} \right)=E\left( \tau_{II,Mo}^{2} \right)=E\left( \tau_{II,Fa}^{2} \right)=p$
- $\mathrm{Var}\left( \tau_{I,Mo} \right)=\mathrm{Var}\left( \tau_{I,Fa} \right)=\mathrm{Var}\left( \tau_{II,Mo} \right)=\mathrm{Var}\left( \tau_{II,Fa} \right)=p-p^{2}=pq$

Then, following standard notation, let $D$ be the covariance between gametes at the two loci. (e.g. Sved, 1968)

The means of cross-products are:

- $E\left( \tau_{I,Mo}\tau_{II,Mo} \right)=E\left( \tau_{I,Fa}\tau_{II,Fa} \right)=D+p^{2}$

By the independence of maternal and paternal gametes:

- $E\left( \tau_{I,Mo}\tau_{II,Fa} \right)=E\left( \tau_{I,Fa}\tau_{II,Mo} \right)=E\left( \tau\right)^{2}=p^{2}$
- $E\left( \tau_{I,Mo}\tau_{I,Fa}\tau_{II,Mo}\tau_{II,Fa} \right)=E\left( \tau_{I,Mo}\tau_{II,Mo} \right)E\left( \tau_{I,Fa}\tau_{II,Fa} \right)=\left( D+p^{2} \right)^{2}$
- $E\left( \tau_{I,Mo}\tau_{I,Fa}\tau_{II,Mo} \right)=E\left( \tau_{I,Fa} \right)E\left( \tau_{I,Mo}\tau_{II,Mo} \right)=p\left( D+p^{2} \right)$

## Correlation between additive codings

- Let the additive coding at locus I be $X_{AI}=\tau_{I,Mo}+\tau_{I,Fa}$ and define $X_{AII}$ analogously for locus II
- Then $E\left( X_{AI} \right)=E\left( X_{AII} \right)=2p$ and $\mathrm{Var}\left( X_{AI} \right)=\mathrm{Var}\left( X_{AII} \right)=2pq$
- The covariance between loci is

$$\begin{matrix} E\left( X_{AI}X_{AII} \right)-E\left( X_{AI} \right)E\left( X_{AII} \right) & =E\left( \tau_{I,Mo}\tau_{II,Mo}+\tau_{I,Mo}\tau_{II,Fa}+\tau_{I,Fa}\tau_{II,Mo}+\tau_{I,Fa}\tau_{II,Fa} \right)-4p^{2} \\ & =\left( D+p^{2} \right)+p^{2}+p^{2}+\left( D+p^{2} \right)-4p^{2} \\ & =2D \end{matrix}$$

- This means that the correlation is $r=\frac{2D}{2pq}=\frac{D}{pq}$, which is the typical correlation coefficient used to summarize LD

## Correlation between parent-of-origin codings

- Let the parent-of-origin coding at locus I be $X_{\gamma I}=\tau_{I,Mo}-\tau_{I,Fa}$ and define $X_{\gamma II}$ analogously for locus II.
- Then $E\left( X_{\gamma I} \right)=p-p=0=E\left( X_{\gamma II} \right)$
- $\mathrm{Var}\left( X_{\gamma I} \right)=E\left( \tau_{I,Mo}^{2}-2\tau_{I,Mo}\tau_{I,Fa}+\tau_{I,Fa}^{2} \right)=p-2p^{2}+p=2pq=\mathrm{Var}\left( X_{\gamma II} \right)$, which is also the same variance as in the additive coding.
- The covariance is:

$$\begin{matrix} E\left( X_{\gamma I}X_{\gamma II} \right)-E\left( X_{\gamma I} \right)E\left( X_{\gamma II} \right) & =E\left( \tau_{I,Mo}\tau_{II,Mo}-\tau_{I,Mo}\tau_{II,Fa}-\tau_{I,Fa}\tau_{II,Mo}+\tau_{I,Fa}\tau_{II,Fa} \right)-0 \\ & =\left( D+p^{2} \right)-p^{2}-p^{2}+\left( D+p^{2} \right) \\ & =2D \end{matrix}$$

- This means that the correlation is also $r=\frac{2D}{2pq}=\frac{D}{pq}$

## Correlation between dominance codings

- Let the dominance coding at locus I be $X_{\Delta I}=2pX_{AI}-2\tau_{I,Mo}\tau_{I,Fa}=2p\tau_{I,Mo}+2p\tau_{I,Fa}-2\tau_{I,Mo}\tau_{I,Fa}$ and define $X_{\Delta II}$ analogously for locus II.
- Then $E\left( X_{\Delta I} \right)=2p^{2}+2p^{2}-2p^{2}=2p^{2}=E\left( X_{\Delta II} \right)$
- The variance is:

$$\begin{matrix} \mathrm{Var}\left( X_{\Delta I} \right) & =EX_{\Delta I}^{2}-E{X_{\Delta I}}^{2} \\ & =E\left( 4p^{2}\tau_{I,Mo}^{2}+4p^{2}\tau_{I,Fa}^{2}+8p^{2}\tau_{I,Mo}\tau_{I,Fa}-8p\tau_{I,Mo}^{2}\tau_{I,Fa}-8p\tau_{I,Mo}\tau_{I,Fa}^{2}+4\tau_{I,Mo}^{2}\tau_{I,Fa}^{2} \right)-4p^{4} \\ & =4p^{3}+4p^{3}+8p^{4}-8p^{3}-8p^{3}+4p^{2}-4p^{4} \\ & =4p^{4}-8p^{3}+4p^{2} \\ & =4p^{2}\left( p^{2}-2p+1 \right) \\ & =4p^{2}q^{2} \\ & =\mathrm{Var}\left( X_{\Delta II} \right) \end{matrix}$$

- - , which is the square of the variance of the additive coding
- The covariance is:

$$\begin{matrix} \mathrm{Cov}\left( X_{\Delta I}, X_{\Delta II} \right) & =E\left( X_{\Delta I}X_{\Delta II} \right)-E\left( X_{\Delta I} \right)E\left( X_{\Delta II} \right) \\ & =E\left( \left( 2p\tau_{I,Mo}+2p\tau_{I,Fa}-2\tau_{I,Mo}\tau_{I,Fa} \right)\left( 2p\tau_{II,Mo}+2p\tau_{II,Fa}-2\tau_{II,Mo}\tau_{II,Fa} \right) \right)-4p^{4} \\ & =E\left( 4p^{2}\tau_{I,Mo}\tau_{II,Mo}+4p^{2}\tau_{I,Mo}\tau_{II,Fa}-4p\tau_{I,Mo}\tau_{II,Mo}\tau_{II,Fa} \right) \\ & +E\left( 4p^{2}\tau_{I,Fa}\tau_{II,Fa}+4p^{2}\tau_{I,Fa}\tau_{II,Mo}-4p\tau_{I,Fa}\tau_{II,Fa}\tau_{II,Mo} \right) \\ & +E\left( -4p\tau_{I,Fa}\tau_{I,Mo}\tau_{II,Mo}-4p\tau_{I,Fa}\tau_{I,Mo}\tau_{II,Fa}+4\tau_{I,Mo}\tau_{II,Mo}\tau_{I,Fa}\tau_{II,Fa} \right)-4p^{4} \\ & =4p^{2}\left( D+p^{2} \right)+4p^{4}-4p^{2}\left( D+p^{2} \right) \\ & +4p^{2}\left( D+p^{2} \right)+4p^{4}-4p^{2}\left( D+p^{2} \right) \\ & -4p^{2}\left( D+p^{2} \right)-4p^{2}\left( D+p^{2} \right)+4\left( D+p^{2} \right)^{2}-4p^{4} \\ & =4p^{4}-8p^{2}\left( D+p^{2} \right)+4\left( D+p^{2} \right)^{2} \\ & =4\left( p^{2}-\left( D+p^{2} \right) \right)^{2} \\ & =4D^{2} \end{matrix}$$

- This means that the correlation is $r_{\Delta}=\frac{4D^{2}}{4p^{2}q^{2}}=\frac{D^{2}}{p^{2}q^{2}}$, which is the square of the LD correlation between locus I and locus II

## Cross-locus, cross-coding correlation: additive and parent-of-origin

- This is relatively simple because the two codings have the same variance and the parent-of-origin coding has a mean of 0

$$\begin{matrix} \mathrm{Cov}\left( X_{AI}, X_{\gamma II} \right) & =E\left( \left( \tau_{I,Mo}+\tau_{I,Fa} \right)\left( \tau_{II,Mo}-\tau_{II,Fa} \right) \right) \\ & =E\left( \tau_{I,Mo}\tau_{II,Mo}-\tau_{I,Mo}\tau_{II,Fa}+\tau_{I,Fa}\tau_{II,Mo}-\tau_{I,Fa}\tau_{II,Fa} \right) \\ & =\left( D+p^{2} \right)-p^{2}+p^{2}-\left( D+p^{2} \right) \\ & =0 \end{matrix}$$

- Because the covariance is 0, the correlation is as well

## Cross-locus, cross-coding correlation: additive and dominance

$$\begin{matrix} \mathrm{Cov}\left( X_{AI}, X_{\Delta II} \right) & =E\left( \left( \tau_{I,Mo}+\tau_{I,Fa} \right)\left( 2p\tau_{II,Mo}+2p\tau_{II,Fa}-2\tau_{II,Mo}\tau_{II,Fa} \right) \right)-4p^{3} \\ & =E\left( 2p\tau_{I,Mo}\tau_{II,Mo}+2p\tau_{I,Mo}\tau_{II,Fa}-2\tau_{I,Mo}\tau_{II,Mo}\tau_{II,Fa}+2p\tau_{I,Fa}\tau_{II,Mo}+2p\tau_{I,Fa}\tau_{II,Fa}-2\tau_{I,Fa}\tau_{II,Mo}\tau_{II,Fa} \right)-4p^{3} \\ & =2p\left( D+p^{2} \right)+2p^{3}-2p\left( D+p^{2} \right)+2p^{3}+2p\left( D+p^{2} \right)-2p\left( D+p^{2} \right)-4p^{3} \\ & =0 \end{matrix}$$

## Cross-locus, cross-coding correlation: parent-of-origin and dominance

$$\begin{matrix} \mathrm{Cov}\left( X_{\gamma I}, X_{\Delta II} \right) & =E\left( \left( \tau_{I,Mo}-\tau_{I,Fa} \right)\left( 2p\tau_{II,Mo}+2p\tau_{II,Fa}-2\tau_{II,Mo}\tau_{II,Fa} \right) \right) \\ & =E\left( 2p\tau_{I,Mo}\tau_{II,Mo}+2p\tau_{I,Mo}\tau_{II,Fa}-2\tau_{I,Mo}\tau_{II,Mo}\tau_{II,Fa}-2p\tau_{I,Fa}\tau_{II,Mo}-2p\tau_{I,Fa}\tau_{II,Fa}+2\tau_{I,Fa}\tau_{II,Mo}\tau_{II,Fa} \right) \\ & =2p\left( D+p^{2} \right)+2p^{3}-2p\left( D+p^{2} \right)-2p^{3}-2p\left( D+p^{2} \right)+2p\left( D+p^{2} \right) \\ & =0 \end{matrix}$$

# References

American Thoracic Society (1995) Standardization of spirometry, 1994 update. Am J Respir Crit Care Med 152:107-1136

Boyd A, Golding J, Macleod J, Lawlor DA, Fraser A, Henderson J, Molloy L, Ness A, Ring S, Davey Smith G (2012) Cohort profile: The ’Children of the 90s’-the index offspring of the Avon Longitudinal Study of Parents and Children. Int J Epidemiol 42:111-127

Delaneau O, Coulonges C, Zagury JF (2008) Shape-it: New rapid and accurate algorithm for haplotype inference. *BMC Bioinformatics* 9:540

Fraser A, Macdonald-Wallis C, Tilling K, Boyd A, Golding J, Davey Smith G, Henderson J, Macleod J, Molloy L, Ness A, Ring S (2013) Cohort profile: The Avon Longitudinal Study of Parents and Children: ALSPAC mothers cohort. Int J Epidemiol 42**:**97–110

Marchini J, Howie B, Myers S, McVean G, Donnelly P (2007) A new multipoint method for genome-wide association studies by imputation of genotypes. Nat Genet 39**:**906–913

Sayers A, Timpson N, Sattar N, Deanfield J, Hingorani A, Davey Smith G, Tobias J (2010) Adiponectin and its association with bone mass accrual in childhood. J Bone Miner Res 25:2212-2220

Sequeira M, Lewis S, Bonilla C, Davey Smith G, Joinson C (2017) Association of timing of menarche with depressive symptoms and depression in adolescence: Mendelian randomisation study. Br J Psychiatry 210:39-46

Sved JA (1968) The stability of linked systems of loci with a small population size. Genetics 59:543-563
